# Decidual macrophages and Hofbauer cells in fetal growth restriction

**DOI:** 10.3389/fimmu.2024.1379537

**Published:** 2024-06-28

**Authors:** Romy Elisa Bezemer, Marijke M. Faas, Harry van Goor, Sanne Jehanne Gordijn, Jelmer R. Prins

**Affiliations:** ^1^ Department of Obstetrics and Gynecology, University Medical Center Groningen, Groningen, Netherlands; ^2^ Department of Pathology and Medical Biology, University Medical Center Groningen, Groningen, Netherlands

**Keywords:** fetal growth restriction, maternal-fetal tolerance, placental development, decidual macrophage, Hofbauer cell

## Abstract

Placental macrophages, which include maternal decidual macrophages and fetal Hofbauer cells, display a high degree of phenotypical and functional plasticity. This provides these macrophages with a key role in immunologically driven events in pregnancy like host defense, establishing and maintaining maternal-fetal tolerance. Moreover, placental macrophages have an important role in placental development, including implantation of the conceptus and remodeling of the intrauterine vasculature. To facilitate these processes, it is crucial that placental macrophages adapt accordingly to the needs of each phase of pregnancy. Dysregulated functionalities of placental macrophages are related to placental malfunctioning and have been associated with several adverse pregnancy outcomes. Although fetal growth restriction is specifically associated with placental insufficiency, knowledge on the role of macrophages in fetal growth restriction remains limited. This review provides an overview of the distinct functionalities of decidual macrophages and Hofbauer cells in each trimester of a healthy pregnancy and aims to elucidate the mechanisms by which placental macrophages could be involved in the pathogenesis of fetal growth restriction. Additionally, potential immune targeted therapies for fetal growth restriction are discussed.

## 1 Introduction

Macrophages, one of the major immune cell subsets in the placenta, play a crucial role in placental development, maternal-fetal tolerance and host defense during pregnancy (1). Macrophages of maternal origin are predominantly located in the decidua and are referred to as decidual macrophages (dMφs) (2, 2). Hofbauer cells (HBCs), fetal macrophages, are the primary immune cell type in the villous stroma and are also present in the chorion and amnion (3). Macrophages are capable of sensing changes in their microenvironment and responding accordingly. This provides macrophages with a high degree of phenotypical and functional plasticity, making them key players in immunologically driven events in pregnancy (2).

For maternal macrophages, roughly three immunological states can be distinguished throughout gestation. These different states require specific adaptive capacity of maternal macrophages to facilitate the processes unique to each phase of pregnancy (4, 5). Early pregnancy is characterized by a pro-inflammatory phase that is needed for implantation of the conceptus and early placental development. This is followed by an immunoregulatory phase that starts around week 13 of gestation which is focused at maintaining maternal-fetal tolerance to ensure that the interaction between maternal and fetal tissue does not result in immunological rejection. The third phase is at the end of pregnancy and is marked by a pro-inflammatory response that initiates the onset of labor (4, 5). Fetal immune cells are naturally impaired in terms of pro-inflammatory cytokine and chemokine production, as well as eliciting cytotoxic responses (6, 7). Both the maternal and fetal immune system are thus inherently biased towards tolerance and the preservation of immune balance.

Maladaptation of the maternal or fetal immune system results in diverted immune functionalities, which can subsequently lead to placental developmental defects and disproportionate inflammatory responses against fetal and placental tissue. Dysregulation of dMφs and HBCs specifically have been associated with adverse pregnancy complications like infertility, preeclampsia, spontaneous preterm birth, (recurrent) pregnancy loss, and intrauterine infections (2, 3, 5, 8).

Fetal growth restriction (FGR) is a condition in which the fetus does not reach its intrinsic growth potential (9). The majority of FGR cases result from placental maldevelopment and subsequent placental insufficiency. However, our understanding of the contribution of placental macrophages to FGR remains limited. (10, 11). Given the many roles that macrophages play in placental development and proper functioning, it is likely that dysfunctional dMφs and HBCs contribute to various aspects of the pathogenesis of FGR. With this review, we aim to elucidate the various mechanisms through which placental macrophages could be involved in the pathophysiology of FGR. To do so, we will first address placental development and functioning under physiological conditions, followed by the pathophysiological aspects of the placenta in pregnancies complicated by FGR. Next, we will provide a comprehensive overview of the functionalities of dMφs and HBCs in healthy pregnancies and outline potential mechanisms by which placental macrophages could be involved in the pathogenesis of FGR. Additionally, potential therapies for fetal growth restriction will be discussed.

## 2 Placental development and anatomy

The placenta consists of a maternal part; the basal plate and decidua, and a fetal part; the chorionic and amniotic membranes, and villi (1). Placental development starts with the formation of the blastocyst, consisting of a trophoblast wall that surrounds the blastocystic cavity, which situates the embryoblast (1). Implantation of the blastocyst in the uterus occurs by the formation of extensions from syncytriotrophoblast, the outer layer of the trophoblast wall, that deeply invade into the endometrium (1). After implantation, the endometrial stromal cells transform into decidual cells forming the decidua (12). Outgrows of the syncytriotrophoblast form the villi with its fetal capillaries, and the intervillous space in between (1).

**Figure 1:**
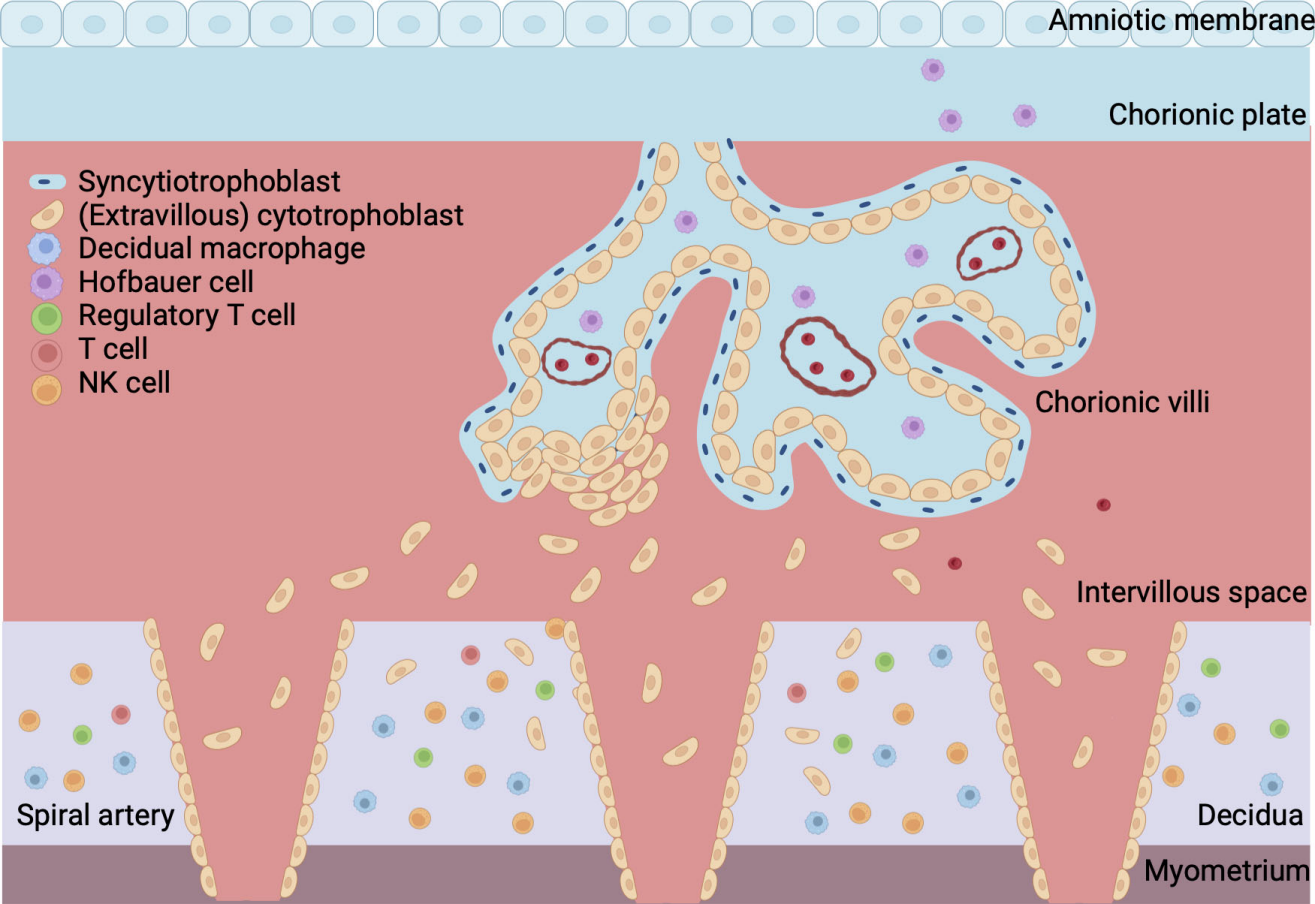
Overview of maternal-fetal interface. The fetal part of the placenta consists of the chorionic plate, amniotic membrane and villi. The cytotrophoblast forms the inner layer of the villi and is surrounded by a continuous outer layer of syncytiotrophoblast. Syncytiotrophoblast cells invade the endometrium during implantation of the conceptus. Extravillous trophoblast invades the maternal spiral arteries to facilitate remodeling of the vasculature. The maternal part of the placenta is formed by the decidua, which is attached to the myometrium, a layer of uterine smooth muscle cells. Blood from the maternal spiral arteries flows into the intervillous space where exchange of oxygen, nutrients and waste between mother and fetus takes place. Created with BioRender.com.

Extravillous trophoblasts (EVT) further invade into the decidua where they destroy and replace the muscular wall of the maternal arteries, a process called spiral artery (SA) remodeling. This creates low resistance, high capacity and low velocity vessels (13, 14). Early in the first trimester, EVT plugs block the SAs in order to prevent a sudden high velocity blood flow into the placenta. The onset of the uteroplacental circulation is marked by the dissolvement of the EVT plugs at the end of the first trimester so that maternal blood can flow into the intervillous space. Hereafter, SA remodeling extends further into the decidua until the inner third of the my (15). **Figure 1** gives a gross anatomical overview of the maternal-fetal interface once placental development is complete.

## 3 Pathophysiology of fetal growth restriction

FGR affects a considerable number of pregnancies, with a prevalence reaching up to ten percent (16). It is associated with a range of perinatal complications including respiratory distress syndrome, sepsis, thrombocytopenia and (iatrogenic) preterm birth (17, 18). Moreover, nearly one third of stillbirths is related to FGR (19). Also, long term consequences like neurodevelopmental disorders and an increased risk for cardiovascular and metabolic diseases later in life can result from restricted growth *in utero* (20–22). The etiology of FGR is multifactorial, encompassing fetal, maternal and placental factors. In the absence of fetal congenital abnormalities, placental malfunctioning is the most common underlying mechanism (10, 11). Typical placental lesions that are observed in the FGR placenta include maternal vascular malperfusion (MVM), fetal vascular malperfusion (FVM), and villitis of unknown etiology (VUE) (9, 23). Often, there is a combination of these lesions (11, 23, 24). MVM, resulting from significant impairments in SA remodeling, is the most common cause for deficits in the uteroplacental circulation (10, 11). It is marked by fewer extravillous trophoblasts (EVTs) that invade the SAs due to increased trophoblast apoptosis, resulting in insufficient disruption of the vascular smooth muscle cell (VSMC) layer with persistent muscularization of the SAs, less fibrinoid deposition in the SA vascular wall and an overall narrow lumen of the vessels with vasoactive capacity. As a consequence, a high velocity, low volume circulation arises which causes mechanical harm like vascular shear stress and damage to the villous structure. Moreover, the alterations in placental blood flow can lead to an increase in oxygen tension and ischemia-reperfusion injury due to intermittent blood flow. Reintroduction of oxygen after deprivation can cause an increase in the formation of reactive oxygen species (ROS), causing oxidative stress and inflammation (11, 25). Placental infarcts arise and villous development is impaired, resulting in villous hypoplasia (26–28). FVM is marked by impaired fetal blood flow, for example due to a high umbilical cord coiling index or vascular changes with fibromuscular muscular sclerosis, intramural fibrin deposition or calcifications leading to thrombosis, and avascular villi, or villous stromal vascular karyorrhexis. Histological features of FVM can be seen in the umbilical and chorionic vessels and stem and terminal villi (29, 30). FGR placentas exhibit characteristic features of oxidative stress and reduced antioxidant protection, resulting in further damage to the villi and impaired transport across the placental barrier (25, 31–33). Another placental lesion commonly found in FGR is VUE, a noninfectious chronic inflammatory lesion of the villi of which the etiology remains poorly understood (34). It has been hypothesized that VUE involves failed maternal-fetal tolerance as the inflammatory infiltrate constitutes of maternal T cells and macrophages that migrate into the intervillous space. In addition, numbers of intravillous HBCs are increased (35).

**Figure 2:**
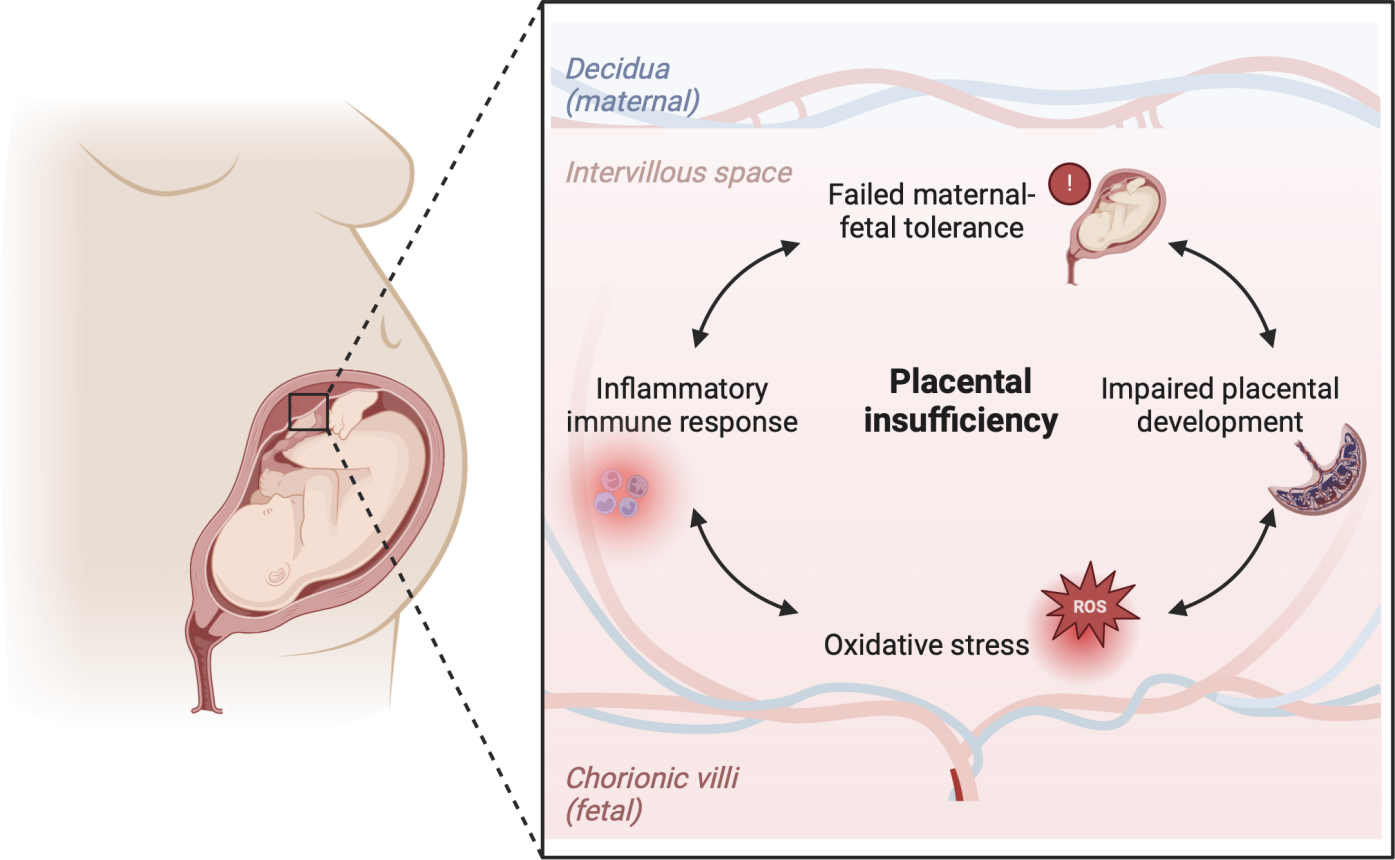
Hypothesis on the immunological etiology of FGR. The hypothesis encompasses a vicious circle of failed-maternal fetal tolerance, placental maldevelopment, oxidative stress and a placental inflammatory immune response. All factors in the cycle influence one another bidirectionally, ultimately causing placental insufficiency, which lies at the root of FGR. Created with BioRender.com.

Failed maternal-fetal tolerance has been proposed as one of the causes of FGR (36). Indeed, several of the above mentioned placental pathophysiological mechanisms can result from, or initiate, a maternal inflammatory response. Multiple studies in FGR placentas have shown increased apoptosis of trophoblast cells as a result of inflammation, which leads to restricted trophoblast invasiveness during implantation of the conceptus and impaired remodeling of the SAs (37–40). On the other hand, maldevelopment of the uteroplacental circulation can cause oxidative stress, which has been implicated in cytokine signaling and regulation of the inflammatory immune response, including the proliferation and pro-inflammatory polarization of macrophages in the placenta (32, 33). Moreover, damage to the placental vascularization and villous tissue can directly elicit a pro-inflammatory response in the placenta. The release of trophoblast fragments into the maternal circulation provides opportunity for maternal immune cells to recognize these fetal antigens and elicit an inflammatory response which compromises maternal-fetal tolerance (37, 41, 42). This interplay can initiate a vicious cycle, as increased oxidative stress attracts more immune cells, potentially exacerbating inflammation and further impairing placental development and functioning (**Figure 2**). Indeed, increased levels of pro-inflammatory markers and damage-associated molecular patterns (DAMPs) can be found in the placenta and in the maternal blood in FGR pregnancies (36). Gene profiling studies on FGR placentas show differentially expressed genes associated with hypoxia, angiogenesis, vascular function, immune modulation and tissue growth in FGR (43–45).

In the next sections, we will outline the functionalities of dMφs and HBCs in healthy pregnancies, followed by reviewing what is currently known about the role of dMφs and HBCs in FGR.

## 4 Decidual macrophages in healthy pregnancy

In early pregnancy, dMφs form up to 20% of the decidual leukocyte population, being the second largest group of immune cells following natural killer (NK) cells (around 70%). T cells account for less than 20% of the decidual immune cell population (46, 47). As mentioned, dMφs have distinct functionalities in each trimester of pregnancy which are enabled by their phenotypical plasticity (4, 5). DMφs numbers increase as pregnancy progresses, highlighting the importance of dMφs throughout the entire course of gestation and making them an interesting cell population to study in healthy and pathological pregnancies (48).

**Figure 3:**
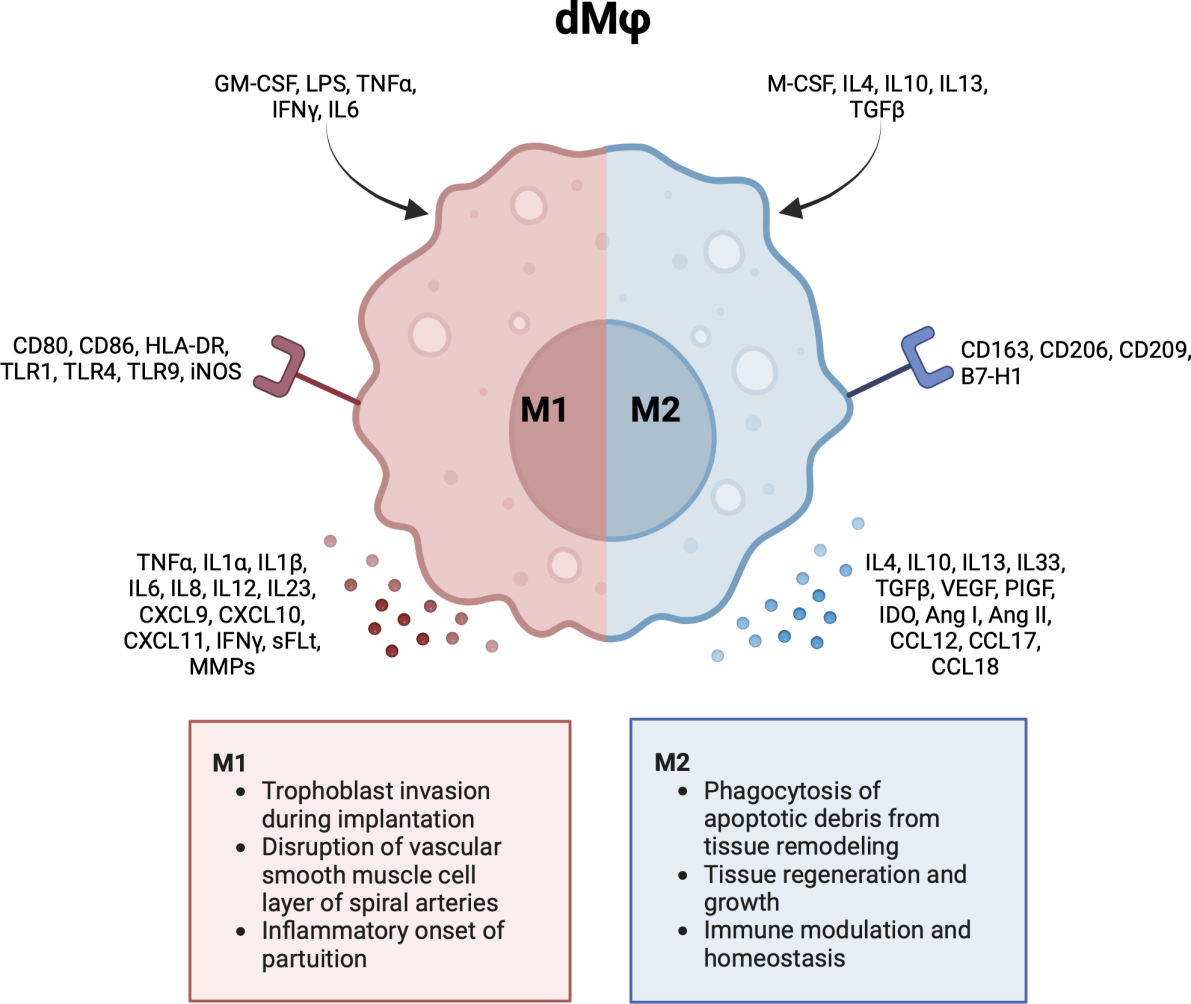
Characteristics and functionalities of decidual macrophages. A simplified classification divides decidual macrophages in M1 and M2 subsets. Polarization factors, cell surface markers and cytokine and chemokines are shown for each subset. Ang, angiotensin; CCL, chemokine (CC-motif) ligand; CD, cluster of differentiation; CXCL, chemokine (CXC-motif) ligand; GM-CSF, growth macrophage colony stimulating factor; HLA, human leukocyte antigen; IDO, indoleamine 2,3-dioxygenase; IFN, interferon; IL, interleukin; LPS, lipopolysaccharide; M-CSF, macrophage colony stimulating factor; MMP, metalloproteinase; PlGF, placental growth factor; sFLT, soluble fms-like tyrosine kinase; TGF, tumor growth factor; TLR, toll like receptor; TNF, tumor necrosis factor; VEGF, vascular endothelial growth factor. Created with BioRender.com.

In general, Mφs can be grouped into classically activated, pro-inflammatory (M1) macrophages, and alternatively activated, anti-inflammatory (M2) macrophages (49). M1 macrophages can be characterized by cell surface markers like cluster of differentiation (CD) 80, CD86 and HLA-DR. M2 macrophages express markers like CD163, CD206 and CD209 (2, 5, 50, 51). Notably, this division represents the extreme ends of a broad dMφ polarization spectrum. It has been proposed to further group M2 dMφs into M2a, M2b and M2c based on the expression of CD11c and HLA-DR (46). However, this division is less often reported for dMφs compared to HBCs. As will be described in the next sections, the majority of dMφs express various phenotypes and functionalities and cannot be strictly classified as M1 or M2. **Figure 3** summarizes the activation pathway, cell surface markers, cytokine and chemokine profiles, and main functionalities of dMφs during pregnancy (2, 5, 47, 50–53).

### 4.1 First trimester

Macrophages are already present in the uterus before pregnancy and play a crucial role in all phases of the menstrual cycle (54). Part of the dMφs are derived from maternal blood monocytes and are recruited to the uterus shortly after conception, which occurs in direct response to semen-derived immune regulatory proteins like MCP-1. Also the embryo itself produces MCP-1 to attract monocytes to the uterus (55). Monocytes migrate towards the syncytiotrophoblast that invades the endometrial stroma during implantation. This migration is guided by trophoblast cells that excrete several signaling molecules (e.g. IL8, MCP1 and MIPα) (56–59). As mentioned in the introduction section, a short pro-inflammatory phase is needed during implantation as this helps trophoblast cells to gain space in the endometrium and prepares the endometrium for implantation of the embryo (57, 58, 60). In response to trophoblast signals, recruited macrophages express pro-inflammatory cytokines like IL6, IL8, TNFα and IFNγ (58). DMφs actively prevent apoptosis of invading trophoblast, for instance by secreting TGFβ which can inhibit NK cells from inducing trophoblast apoptosis, and IL6 and IL10 which can downregulate Fas mediated apoptosis (61, 62). DMφs also secrete G-CSF and IL33, which promote trophoblast proliferation, migration and invasion (63, 64). Conversely, several *in vitro* studies have shown that pro-inflammatory monocytes can restrict trophoblast invasiveness and increase trophoblast apoptosis (65–68). This suggests that the pro-inflammatory macrophage response needs to be balanced and carefully timed to prevent trophoblast damage.

In mice, it has been clearly demonstrated that macrophages have pro-inflammatory characteristics early in the implantation phase, followed by a mixed pro-inflammatory and immunomodulatory response once trophoblast further invades the uterine stroma and placental development continues (55). In humans, a similar shift in dMφs phenotype can be expected in the first trimester. Indeed, some studies in humans describe both anti- and pro-inflammatory characteristics in first trimester dMφs (69–72). For example, macrophage activation markers like HLA-DR, CD80 and CD86, involved in macrophage T-cell interactions, can be found on first trimester dMφs (69, 70). Others found dMφs displaying immunomodulatory phenotypical markers (CD163, CD206 and CD209) to be capable of secreting both anti- and pro-inflammatory cytokines (71, 72). However, the majority of studies indicate dMφs in humans to be predominantly anti-inflammatory and immunomodulatory. Distinct observations on the phenotype of dMφs are likely due to differences in the specific time point in the first trimester at which the experiments are performed. Studies report expression of anti-inflammatory markers (e.g. CD163, CD206 and CD209) and the secretion of anti-inflammatory cytokines (e.g. IL10, CCL12, CCL17 and CCL18) (69, 72–75). Gene expression profiling studies show that genes related to immunomodulation and tissue remodeling are activated in first trimester dMφs (76). This is further observed when studying the response of dMφs to placental signals. Multiple studies have cultured dMφs with placental explants or with decidua or trophoblast derived cytokines and demonstrate that polarizes dMφs towards a more anti-inflammatory phenotype (73, 74, 77, 78). It was even shown that HLA-G expressed on invading trophoblast can directly bind to leukocyte immunoglobulin-like receptors on dMφs to induce a more anti-inflammatory phenotype in dMφs (79–82).

Besides implantation, dMφs participate in SA remodeling (83). The migration of dMφs towards the SAs relies on crosstalk between trophoblast cells and SA endothelial cells (ECs). Several factors expressed by EVT (e.g. CXCL8 and IL6) stimulate the ECs to secrete CCL14 and CXCL6, causing dMφs to home to the walls of the SAs. At this site, dMφs secrete several cytokines (e.g. IL1β, IL6, IL8, IL10 and IFNγ) that are involved in remodeling of the SAs (84). DMφs cause disruption of the VSMC through the expression of several metalloproteinases (MMP) (85, 86) and induce apoptosis and phagocytosis of VSMCs (87, 88). The apoptotic cell bodies that are released into the maternal circulation during placental development are cleared by dMφs, which is accompanied by the release of anti-inflammatory cytokines (88). Furthermore, dMφs exert angiogenic properties via the production of VEGF and angiogenic growth factor (Ang) 1 and Ang2 (50, 89, 90). Similar to implantation of the embryo, remodeling of SAs seems to require a short inflammatory phase. In the SAs, this is characterized by foamy (lipid filled) macrophages in the intima of the vessel wall that resolve once SA remodeling is complete (90). It is important that this inflammatory response is transient as persistent inflammation can cause acute atherosis, marked by fibrinoid necrosis of the vessel wall, lymphocytic infiltration, and excessive accumulation of foamy macrophages, potentially resulting in MVM (84, 91). Besides alterations in dMφ activation, dysregulation in their numbers can cause defects in placental development. A macrophage surplus can lead to excessive apoptosis of EVT, limiting the invasion of trophoblast in the SAs and causing vessels to be (partly) unremodeled (88, 92). Mouse studies have shown that also reduced macrophage numbers can result in suboptimal implantation, hypocellularity of the decidua and a thickened VSMC layer in decidual maternal blood vessels, indicating impaired decidualization and incomplete remodeling of the uterine vasculature (93, 94).

Next to their role in placental development, first trimester dMφs are involved in maintaining a tolerogenic intrauterine environment. DMφs are located in close proximity to decidual Treg cells and can induce Treg cell expansion through binding of CD80 and CD86 on dMφs to cytotoxic T-lymphocyte antigen 4 (CTLA4) on Treg cells. At the same time, this interaction increases the activity of indoleamine 2,3-dioxygenase (IDO) in dMφs, an enzyme known to induce immune tolerance in various immune cell subsets. In pregnancy, IDO is involved in preventing a harmful maternal T cell response towards the fetus (95). Another mechanism of preventing a maternal inflammatory response is through interaction of programmed death (PD)1 on T cells with its ligand (PDL1) on dMφs leading to downregulation of the IFNγ production by maternal T cells (96).

From the studies on first trimester dMφs we can conclude that their numbers and phenotypical characteristics must be carefully regulated to promote trophoblast viability and invasiveness, processes that are important for implantation of the embryo and remodeling of the SAs. Both implantation and SA remodeling are facilitated by a short inflammatory dMφ response important for trophoblast invasiveness, followed by dMφs that predominantly express immunomodulatory characteristics to further support placental development and maternal-fetal tolerance.

### 4.2 Second trimester to term

During the second trimester of pregnancy, numbers of dMφs continue to expand to nearly half of the maternal immune cell population at mid-pregnancy (46). Most of these second trimester dMφs are positive for anti-inflammatory, immunoregulatory cell surface markers like CD163, CD206, and CD209 (46, 97). Interestingly, HLA-DR, CD80 and CD86, co-stimulatory molecules for T cell activation, are expressed on second trimester dMφs. These molecules also have the ability to weaken T cell responses by binding to CTLA-4 expressed on decidual T cells, indicating that the expression of co-stimulatory molecules on dMφs might not cause T cell activation in pregnancy (97). Indeed, it was shown that dMφs co-cultured with allogeneic T cells were unable to induce T cell proliferation or activation, and instead caused upregulation of Treg cells (98). One mechanism for promoting Treg cell activity was found to be through the production of cellular retinal dehydrogenase by dMφs that can synthesize retinoic acid, which regulates Treg cells (99). In addition, dMφs suppress cytotoxic NK and T cell activity through TIM3 and Galectin9 expression (46).

Third trimester dMφs are high in CD163 expression and relatively low in CD80, CD86 and HLA-DR (99, 100). In addition, genes encoding for a pro-inflammatory macrophage phenotype are silenced, while genes for a more regulatory, immunotolerant phenotype are activated (101). Similar to the first trimester, the decidua itself is key in promoting the immunoregulatory macrophage phenotype. Upregulation of CD163 and CD209 and downregulation of CD86 has been observed after conditioning monocytes with cell medium from term decidual tissue (102). In mice, pro-inflammatory immune responses can be downregulated by decidua derived progesterone through inhibition of toll like receptor (TLR) 4 and TLR9 triggered release of IL6 and nitric oxide (NO) by dMφs (103). Another mechanism proposed to induce maternal-fetal tolerance is through exposure of maternal macrophages to trophoblast debris. Phagocytosis of trophoblast cells from term human placental explants by maternal blood monocytes resulted in reduced expression of CD80, CD86, CD40, MCP1, and B7-H3 (involved in suppressing T cell activation), increased secretion of cytokines like IL1β, IL12p70 and IL8, and upregulation of IDO (104).

Near birth, the immunoregulatory role of dMφs changes as dMφs participate in the inflammatory response that triggers the spontaneous onset of labor. This is marked by an increased level of inflammatory cytokines like IL1ß, IL8 and IL6 during labor, as well as elevated numbers of dMφs with pro-inflammatory character (105, 106). One study even reported macrophages to increase up to four times in laboring women (107). In rats, it could be demonstrated that macrophages infiltrate the decidua prior to the onset of labor, further suggesting that dMφs directly initiate parturition (107). To summarize, the second and third trimester of pregnancy, before the spontaneous onset of labor, are focused at immunotolerance and the downregulation of potential pro-inflammatory responses, which safeguards maternal-fetal tolerance and improves placental functioning, allowing for optimal fetal growth. At term, dMφs start to display pro-inflammatory characteristics that promote the onset of labor.

## 5 Hofbauer cells in healthy pregnancy

**Figure 4:**
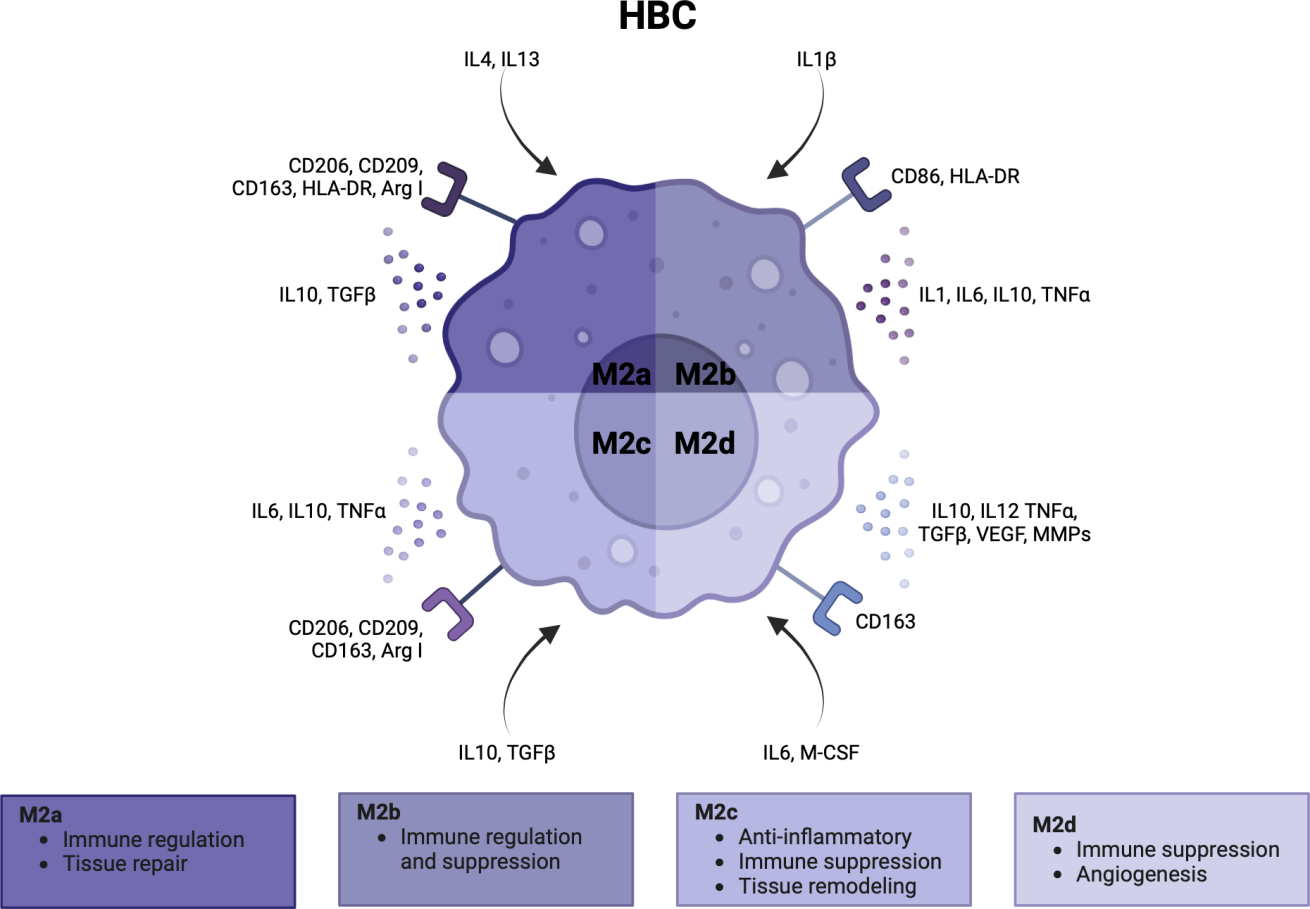
Characteristics and functionalities of Hofbauer cells. A simplified classification divides Hofbauer cells in M2a, M2b, M2c and M2d subsets. Polarization factors, cell surface markers and cytokine and chemokines are shown for each subset. Arg, arginase; CCL, CD, cluster of differentiation; HLA, human leukocyte antigen; IL, interleukin; M-CSF, macrophage colony stimulating factor; MMP, metalloproteinase; TGF, tumor growth factor; TNF, tumor necrosis factor; VEGF, vascular endothelial growth factor. Created with BioRender.com.

HBCs form over 90% of the fetal immune cell population in the placental villi. They appear at day 18 of pregnancy, increase up until 18 weeks and decrease as pregnancy progresses (1, 3, 108–110). Early in pregnancy, HBCs stem from primitive yolk sac macrophages that proliferate in the villi or from villous mesenchymal stem cells (1, 111, 112). Once the fetal circulation has been established, HBCs differentiate from fetal monocytes in the bone marrow that are recruited to the placenta (3) (113, 114). Similar to dMφs, the polarity of HBCs can be simplified as M1 and M2, although HBCs generally lack the M1 phenotype. Some studies subdivide M2 HBCs into M2a, M2b, M2c and M2d (8, 115). M2a macrophages are seen as ‘alternatively activated’ macrophages that are involved in immune regulation and tissue repair. M2b macrophages suppress and regulate inflammation as they can exert Th2 responses and produce a range of pro- and anti-inflammatory cytokines. M2c have more anti-inflammatory and tissue remodeling capacities, and M2d macrophages are involved in immune suppression and angiogenesis (49, 112, 115, 116). In **Figure 4**, the activation pathway, cell surface markers, cytokine and chemokine profiles, and main functionalities of HBCs in pregnancy are summarized (3, 5, 112, 117).

### 5.1 First trimester

In the first trimester, the large majority of HBCs are of the M2a (CD206^+^CD209^+^HLA-DR^+^CD163^+^) and M2c (CD206^+^CD209^+^HLA-DR^-^CD163^+^) subtype which are involved in tissue repair and immunoregulation. These cells express arginase (Arg) I and Arg II, enzymes involved in M2 polarization (110, 111). M1 markers like CD80, CD86 or iNOS, an enzyme involved in the M1 polarization pathway, are not expressed (110). Findings on the expression of HLA on first trimester HBCs are conflicting. While the majority of studies report HBCs to be typically low in HLA in the first trimester (111, 115, 118–120), some have found clusters of M1-like or M2a/M2b HBCs that do express the classical HLA-DR (110, 115).

Several alternative subdivisions of HBC types have been suggested. For example, classification into a group with an anti-inflammatory profile (IL11, IL17, TGFβ and VEGF) that formed the vast majority of HBCs, and a pro-inflammatory group that constituted only 5% of the population (IL1β, IL6 and TNFα) (121). Another division based on the expression of CD74 (gamma chain of HLA class II) revealed a CD74^+^ HBC subset that expressed genes encoding for cytokine stimuli, inflammatory responses, antigen presentation and myeloid leukocyte activation. This subset was suggested to be involved in removal of cellular debris during early development of the placenta (122). Another mechanism for the removal of anti-fetal antibodies from the placenta is through the expression of three subtypes of the Fc receptors for IgG (FcγR), FcγRI (CD64), FcγRII (CD32) and FcγRIII (CD16) on HBCs that can bind antigen-antibody complexes (123). These receptors are furthermore implicated in the transplacental transmission of maternal IgG antibodies into the fetal circulation, providing HBCs with a crucial role in immunological protection of the fetus (8, 123–125). HBCs are furthermore involved in the development of the villi and its vasculature. They are found in abundance within the mesenchymal villous stroma that later develops into immature intermediate villi and are found in close association with fetal capillaries (108, 126, 127). Culturing trophoblast cells with HBC supernatant lead to enhanced trophoblast growth and differentiation (128). HBCs are the primary cells expressing sprouty proteins, important for branching villous and vascular morphogenesis and growth factor signaling (129). Moreover, HBCs produce VEGF and its receptors, and several other factors like fibroblast growth factor 2 (FGF-2) and osteopontin (OPN) that promote placental growth and angiogenesis, and tissue inhibitor metalloproteinase 9 (TIMP-9) and MMP-9 that facilitate trophoblast invasion (111, 130–135). Moreover, HBCs exhibit phagocytic capacities and bactericidal functions by producing reactive oxygen species (ROS) and glucose-6-phosphate dehydrogenase (G6PD) (111, 136).

To conclude, the largest share of the first trimester HBC population is involved in immune regulation and supporting a tolerogenic environment by removing tissue debris and fetal antibodies. In addition, HBCs have an important role in the development of the villous architecture and the fetal vasculature.

### 5.2 Second trimester to term

During the second and third trimester, HBCs are found in all types of villi where they are located close to the fetal capillaries, although they are rarely seen in terminal villi (109, 115, 137–139). Almost no expression of M1 markers like CD40, CD11b and CD80 has been observed at term, while M2 markers like CD163, CD209, CD206, folate receptor (FR)-β are abundantly expressed (110, 115, 140). Genetic profiling of HBCs confirms this as M1 associated genes are silenced, and M2 related genes are activated (101).

In the third trimester, a notable shift in HBC phenotype is observed, characterized by a decrease in M2a and increase in M2c HBCs, which ultimately form the predominant subset (110, 115). M2c HBCs are engaged in anti-inflammatory reactions, tissue remodeling and immune suppression. M2b cells, mostly absent in the first and second trimester, emerge near term and constitute approximately one third of the HBC population (110, 115). They can be identified by CD86 expression, alongside the secretion of IL6 and TNFα. The M2b subset is stimulated by immune complexes and participates in antibody-mediated immunity (110, 115). Consistent with findings in the first trimester, studies report different results regarding HLA expression on HBCs. Either varying expression of HLA-DR between placentas, lack of HLA-DR expression or presence of HLA on the majority of HBCs have been reported (46, 110, 134, 140–143). One hypothesis proposed to explain these discrepancies is the mode of delivery, wherein placentas obtained after primary cesarean section exhibit lower HLA expression due to a lack of inflammatory responses that mark the onset of parturition (138). Alternatively, the presence of HLA on HBCs could indicate a matured third trimester subset that is capable of antigen presentation to T cells (142). This would be in line with the increase of M2b HBCs in the third trimester (110, 115).

Third trimester HBCs are involved in maintaining a state of immune tolerance. HBCs secrete IDO and IL10, which induce an anti-inflammatory phenotype in maternal immune cell subsets like macrophages and Tregs (73, 144). Mixed lymphocyte reactions show that third trimester HBCs inhibit the activation of lymphocytes, and the proliferation and production of cytotoxic cells (145). Limiting potentially harmful immune responses is also established through the expression of death-inducing TNF superfamily ligands and receptors that are implicated in autocrine programmed cell death, and therefore also involved in placental tissue remodeling processes (146). One study showed that third trimester HBCs might be more biased towards immune tolerance compared to HBCs earlier in pregnancy. It was demonstrated that first and second trimester HBCs could be polarized into M1-like cells with higher levels of inflammatory cytokine secretion (e.g. IL1β, IL12, IL6 and TNFα) using LPS, while HBCs at term kept an M1/M2 balance and did not upregulate M1 markers like CD80, CD86, TLR1 and TLR4 in response to LPS (110, 120). Although this response might be lower in the third trimester, studies consistently demonstrate that LPS stimulation can increase the number of HBCs and induce the secretion of inflammatory cytokines like TNFα, IL10, IL1α, IL1β, IL6 and IL12 (121, 139, 141). Term HBCs express low levels of NOS type II, which can implicate a surveilling function to detect pro-inflammatory events or maternal pathogens by being able to respond with a cytotoxic effect through NO secretion (147). DNA methylation studies of HBCs show enrichment of genes involved in immunological defense, inflammation, acute inflammatory response to stress, regulation of immune cell activation, complement activation, cell adhesion and angiogenesis (101).

Third trimester angiogenetic capacities of HBCs are mainly due to VEGF, which is produced at even higher levels in the third trimester compared to the first. VEGF producing HBCs are located near regions where Ang 1 and 2 is expressed within the mature intermediate and terminal villi (133, 134). Together with VEGF, HBCs secrete FGF2, which enhances endothelial branching and chemoattraction of fetal endothelial cells (115). HBCs furthermore contribute to the regulation of placental blood flow through thromboxane and prostaglandin E2, which are involved in vasoconstriction and vasodilatation respectively (148). HBC derived IL17 is likely involved in the upregulation of these angiogenic factors (149).

Taken together, second and third trimester HBCs have similar functionalities compared to the first trimester. From the aforementioned studies we can conclude that a more mature subset of HBCs emerges as pregnancy progresses, which is involved in immunosurveillance and downregulation of potential pro-inflammatory responses. HBCs furthermore continue to participate in the development of the placental vascularity throughout the second and third trimester.

## 6 Macrophages in fetal growth restriction

Numerous studies have emphasized the significance of dMφs and HBCs in placental functioning and the maintenance of an intrauterine environment that is tolerant to the conceptus. Given the diverse array of functions, it comes to no surprise that disturbances in numbers, phenotypes or functionalities of placental macrophages have been observed in various pregnancy complications that are characterized by defects in placental development. These include preeclampsia, spontaneous preterm birth, preterm premature rupture of membranes and (recurrent) pregnancy loss, among others (106, 150–152). For FGR specifically, the mechanisms through which imbalances in placental macrophages contribute to its pathogenesis remain largely unclear due to the limited number of studies on this topic.

We hypothesize the contribution of dMφs and HBCs to the pathophysiology of FGR to be multifaceted. Generally stated, dMφs work on the maternal side of the placenta where they are crucial for embryo implantation and SA remodeling, whereas HBCs are active in the villi, contributing to villous maturation and the formation and growth of the fetal vascularity. In addition, both dMφs and HBCs employ various mechanisms to maintain a tolerogenic intrauterine environment, such as promoting the differentiation of maternal T cells into Treg cells, inhibiting maternal T cell activation and clearing antibody-antigen complexes that could potentially trigger a maternal immune response against the fetus. Close regulation of the number and functionalities of placental macrophages is essential for adequate placentation and the maintenance of maternal-fetal tolerance.

**Figure 5:**
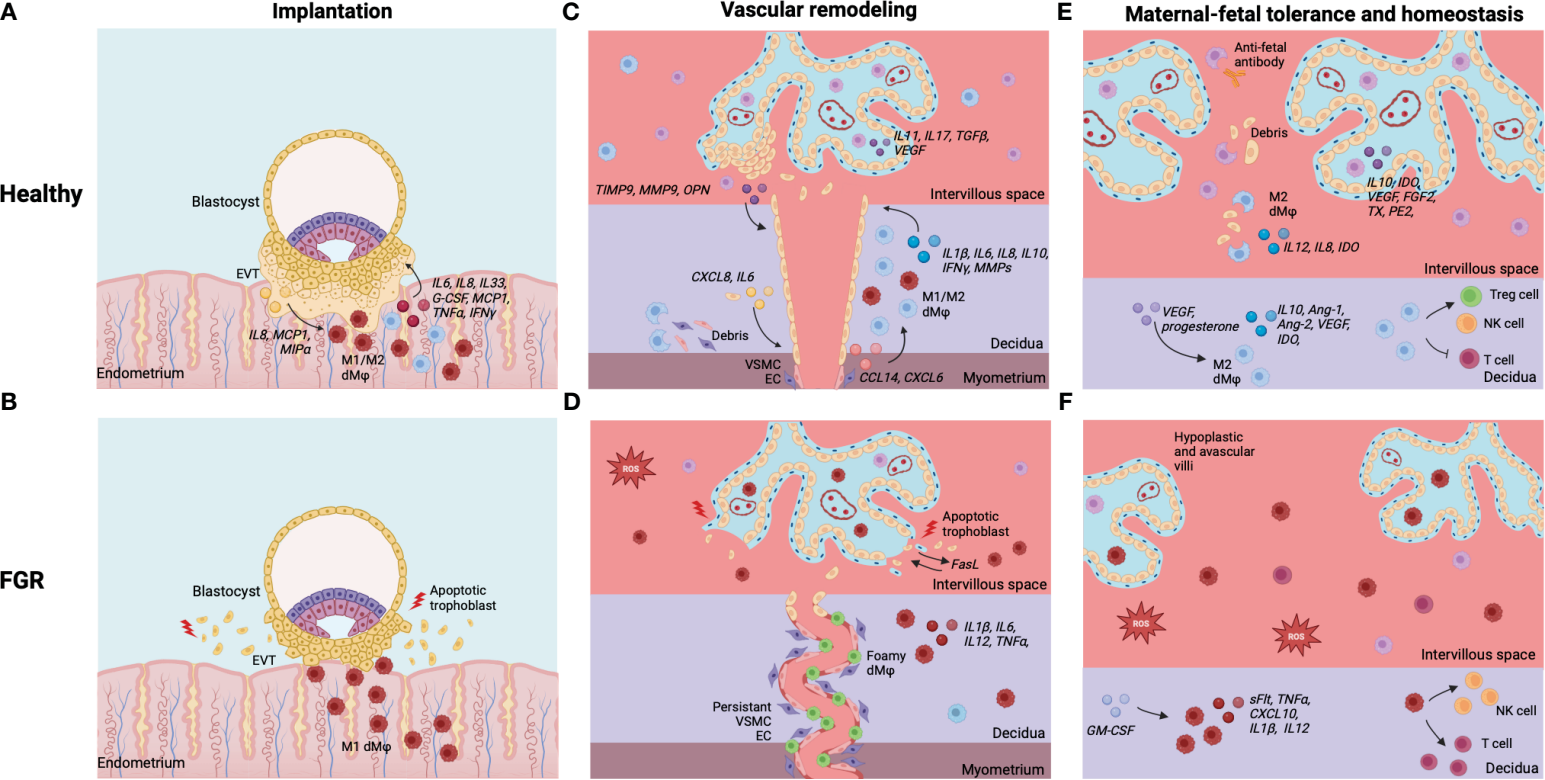
Summary of dMφs and HBCs in healthy pregnancies and FGR. **(A)** Implantation in healthy pregnancies: EVT and dMφs crosstalk facilitates deep invasion of EVT in endometrial stroma for implantation of the embryo. **(B)** Implantation in FGR: excess of inflammatory dMφs and EVT apoptosis, leading to shallow implantation of the embryo. **(C)** Vascular remodeling in healthy pregnancies: dMφs and HBCs cause disruption of VSCM and EC within maternal SA and stimulate EVT invasion, which causes remodeling of the SA into dilated, low-resistance vessels. **(D)** Vascular remodeling in FGR: excess of inflammatory (foamy) dMφs causes apoptosis of EVTs, leading to restricted EVT invasion. Disruption of the VSCM and EC layers is incomplete and the lumen of the SA remains narrow. **(E)** Tolerance and homeostasis in healthy pregnancies: dMφs and HBCs secrete anti-inflammatory cytokines and vascular growth factors, phagocytose placental debris and anti-fetal antibodies, and induce Treg cells while inhibiting potential inflammatory T cell and NK cell responses. **(F)** Tolerance and homeostasis in FGR: balance shifted towards dMφs and HBCs that secrete pro-inflammatory cytokines, lack vascular growth factors, and stimulate T cells and NK cells, leading to an inflammatory and hypoxic environment with impaired placental vascularization and damage to the villi. Created with BioRender.com.

Most likely, disturbances in dMφ and HBC activation could both be a cause and a consequence of placental insufficiency in FGR pregnancies. On one hand, it is possible that initial disruption of maternal-fetal tolerance skews dMφs and HBCs towards a more pro-inflammatory subset, resulting in defects in placental development and disturbed functioning of the uteroplacental circulation throughout pregnancy. On the other hand, placental insufficiency can be caused by a variety of mechanisms. For example, maternal hypertensive disorders, diabetes mellitus, auto-immune diseases, obesity and smoking are all associated with maternal vasculopathy and an activated maternal immune system, which can cause placentation defects (16, 153). In these cases, it could be that the oxidative stress arising from impaired utero-placental perfusion and ischemia-reperfusion injury triggers an inflammatory response that results in pro-inflammatory macrophage polarization. Interestingly, a large number of pregnancies are complicated by placental insufficiency in the absence of factors that predispose to placental maldevelopment. It could be these pregnancies in which impaired maternal-fetal tolerance causes placentation defects in otherwise healthy women. The different etiologies of FGR makes that the interaction between placental functioning and placental macrophage populations in the context of FGR multifaceted and complex. Our overall theory is that placental insufficiency, oxidative stress and inflammation form a vicious circle that contributes to the pathogenesis of FGR, as depicted in **Figure 2**. Understanding this interaction in the pathophysiology of FGR is crucial for unraveling the immunological mechanisms at play. In the next section, the current knowledge on the role of dMφs and HBCs in human and animal pregnancies complicated by FGR will be discussed. **Figure 5** summarizes the functionalities of dMφs and HBCs in healthy pregnancies and FGR.

### 6.1 Decidual macrophages in fetal growth restriction

Previously, we investigated decidual immune cell subsets in term pregnancies affected by FGR and showed a 50% increase in CD68^+^ dMφ numbers in FGR compared to healthy pregnancies, accompanied by decrease of the CD206^+^/CD68^+^ dMφ ratio, suggesting a reduction of anti-inflammatory dMφs in FGR (24). The majority of FGR placentas in our cohort showed signs of VUE and a combination of multiple placental lesions (MVM, FMV and VUE) (24). Contrary to our results, another study showed no differences for dMφ numbers, defined as CD14^+^, between FGR and healthy pregnancies at term. There was no assessment of placental pathology (154). Other studies do confirm our observations. Higher numbers of CD68^+^ dMφ with increased HLA-DR staining were detected in the extravillous areas of term placentas. FGR cases with chronic villitis and VUE were excluded in this cohort (155). Another study showed elevated numbers of CD68^+^ and CD163^+^ macrophages in the intervillous space of FGR pregnancies, likely to be maternal macrophages that migrated there. Placentas showed signs of immaturity of the chorionic villi with sclerosis, intervillous fibrinoid deposition and calcification, resulting in afunctional villous areas. In addition, preterm FGR cases (born before 37 weeks) showed impaired vascularization and villous hypoplasia (156). Another study comparing gene expression between term FGR and control placentas showed more expression of genes for total macrophages and M1-like macrophages in FGR cases, which correlated with NEURL1 and ODF3B gene expression, both involved in immune cell proliferation, activation and inflammation (157).

One study investigated both second and third trimester dMφs in FGR pregnancies. In the second trimester, an increased number of CD68^+^ macrophages was detected in the microlymphatic vessels between decidual cells, in contrast to healthy pregnancies where macrophages are found in the decidual stroma or transforming vessels. This finding was associated with impaired SA remodeling. In third trimester FGR placentas, CD68^+^ macrophages were identified in leukocyte clusters close to apoptotic EVTs. There was a lack of EVT invasion in the SA, along with thickened VSMC layer and intact endothelium, suggesting that a macrophage excess was involved in trophoblast cell death and subsequent impaired SA remodeling (28).

Furthermore, it appears that macrophages from FGR pregnancies have impaired angiogenic capacities. Maternal monocytes differentiated into M2-like macrophages that were cultured with placental mesenchymal stromal cells from FGR pregnancies showed inhibited tube formation in *in vitro* assays, suggesting that macrophages from FGR pregnancies can cause impaired remodeling of the uterine vasculature (158). Although limited, the existing literature points towards an increase in macrophages of a more inflammatory character in FGR, which may have detrimental effects on implantation and the development of the placental vasculature.

### 6.2 Hofbauer cells in fetal growth restriction

One study found CD68^+^ HBCs to be increased in FGR cases compared to healthy pregnancies, while HBCs positive for CD206 were reduced. Placentas with VUE were excluded and no other placental pathology data was available (155). On the contrary, also lower numbers of CD68^+^ and CD163^+^ HBCs have been found in the terminal villi of term and preterm FGR compared to healthy controls. The FGR placentas showed signs of villous immaturity and afunctional villous areas (159). This decrease in CD68^+^ HBCs has also been reported by another study. However, when A1 antichymotrypsine was used as a marker for HBCs, more HBCs were found in FGR (160). The authors suggest that A1 antichymotrypsine, a protein involved in the inhibition of natural killing and antibody-dependent cell-mediated cytotoxicity, is more specific for HBCs than CD68, which could explain the different results. They proposed that the increase in HBCs could be a compensatory mechanism to prevent a maternal immune response towards fetal alloantigens. The placental morphology and histopathology was not assessed in this study (160). The limited availability of studies and the use of different markers makes it difficult to draw conclusions on the role of HBCs in FGR.

### 6.3 Animal studies on macrophages in fetal growth restriction

Different mouse models have been used to mimic FGR and investigate placental immune cell subsets. However, it must be considered that placentation in mice varies from placentation in humans. Mice placentas have a distinct trophoblast cell type that is less invasive compared to humans, and the placental labyrinth structure differs from the more open intervillous space in human placentas (161). Two mouse models, one based on the deficiency of FcR-γ and another one on complement factor C5a receptor deficiency, exhibit high rates of spontaneous pregnancy loss and severe FGR in the surviving fetuses, alongside morphological signs of impaired placental vascularization. There was an increased infiltration of monocytes and neutrophils, along with a functional deficiency of VEGF (162). Another mouse model based on PBX1-deficient decidual NK cells resulted in unexplained recurrent pregnancy loss and FGR. The placentas of these mice showed higher infiltration of dMφs and neutrophils compared to the wild type at mid gestation. No differences in placental morphology was found between FGR and control pregnancies (163). Also mice undergoing BCG-trained immunity show a higher incidence of FGR, but contrary to other studies, show decreased placental macrophages in the second week of pregnancy (similar to second trimester in humans) compared to controls. However, these macrophages did show a tendency towards a more inflammatory profile. The authors hypothesize that a lack of macrophages causes placental developmental defects, such as impaired EVT infiltration and reduced SA remodeling, causing FGR. Unfortunately, this hypothesis was not substantiated with placental morphology assessment at mid pregnancy. At term, no differences in placental morphology were found for FGR pregnancies and numbers of placental macrophages did not differ from control mice (159). Another way to induce FGR in mice is the administration of uric acid, which was found to induce placental inflammation and an increase of CD68^+^ macrophages in the junctional and labyrinth zone. No changes in placental gross histopathology were seen (164). Imbred mice mating models also demonstrate FGR, with a 1.8-fold increase in F4/80^+^ macrophages, localized in the labyrinth and chorionic plate. At term, the macrophage number increased up to 11-fold. The placentas from FGR pregnancies were smaller, displayed signs of thrombosis and fibrosis and a reduced size of the placental labyrinth (165). Inflammation in mice has furthermore shown to impact fetal growth and placental macrophage levels in subsequent generations. First degree offspring of LPS treated female mice showed higher FGR rates compared to controls. Interestingly, their offspring (second generation) showed impaired fetal growth as well, together with higher levels of CD68^+^ macrophages and lower levels of CD163^+^ macrophages in the mesometrial triangle, junctional zone and labyrinth. Deficient trophoblast invasion and narrower SAs were observed and Doppler measurements confirmed inadequate SA perfusion (166). Another study used a mouse model based on maternal KIR2DL1 expression on NK cells and paternal HLA-C*050 expression on fetal trophoblast cells that lead to FGR with impaired remodeling of the SA and high resistance placental blood flow. FGR placentas were enriched in genes for macrophage recruitment and macrophages showed higher expression of genes involved in macrophage invasion under hypoxic conditions, dendritic cell maturation, antigen presentation and inflammation (167). To summarize, these mouse models reveal that FGR placentas can be characterized by elevated numbers of placental macrophages skewed towards a pro-inflammatory phenotype. These immunological alterations likely contribute to the observed disturbances in placental development and functioning.

## 7 Current therapeutic approach for fetal growth restriction

At this point, there is no established and effective therapy to prevent or treat FGR. The current approach involves close monitoring with Doppler ultrasound and cardiotocography (CTG) and timely delivery of the baby (168). Although there are certain maternal serum biomarkers indicative for combined FGR and PE, no diagnostic biomarkers for FGR solely can be found that have additive value to biometry in predicting FGR (169–171).

Women with significant risk for developing FGR are often counseled before or early in pregnancy and monitored throughout (16). For pregnant women with previous pregnancies complicated by FGR and/or PE, or other specific risk factors, acetylsalicylic acid is often prescribed. Some studies have shown that starting acetylsalicylic acid before 16 weeks of pregnancy can significantly reduce the risk of FGR (172, 173). Acetylsalicylic acid has progangiogenic, vasodilatory and antithrombotic effects through upregulation of pro-angiogeneic factors like PIGF, prostacyclin and NO, and improves trophoblast invasion and SA remodeling (174). Moreover, acetylsalicylic acid-triggered lipoxins have anti-inflammatory, antioxidant and immune-modulatory functionalities by inhibiting NF-kB activation and TNF secretion by T cells, and upregulation of IL10 and NO (174, 175).

The combination of acetylsalicylic acid with low molecular weight heparin (LMWH) has also been suggested as a therapeutic option for FGR. LMWH has an anticoagulant effect on the placental vasculature and could furthermore improve endothelial function, increase NO and PlGF, and reduce pro-inflammatory cytokines like IL8, IL6 and TNFα (174–176). Statins too have been explored as an approach to improve placental functioning in FGR and PE. It has potential pro-angiogenic and antioxidant effects, and could aid in endothelial protection and reduction of placental inflammation. However, studies on the use of LMWH or statins are scarce and conflicting as to whether this has beneficial clinical effects (177, 178).

A drug that has been in trial for early-onset FGR is sildenafil, a phosphodiesterase-5 inhibitor suggested to enhance the function of NO and thereby promote vasodilatation of the maternal and fetal vasculature. Yet, no positive effects on placental functioning and the improvement of fetal growth have been observed (179).

### 7.1 Immune targeted therapies

Direct modulation of macrophage phenotype and functionality has not been investigated in the context of pregnancy complications. Potential strategies could be targeted at macrophage polarization, cytokine and chemokine secretion, or signaling pathways by which macrophages interact with other immune cells. Naturally, these macrophage modulators must be safe to use in pregnancy. Several studies have demonstrated that culturing macrophages with medium derived from placental tissue can induce favorable M2-like characteristics in placental macrophages (58, 73, 102). Therefore, investigating the specific placental mediators at play could be promising in finding potential macrophage regulatory therapies. For example, vitamin D is produced by the placenta and can affect macrophage polarization by binding to intracellular vitamin D receptors (VDR) present in macrophages (180). Within the placenta, it stimulates the interaction between placental macrophages and Treg cells and skews the balance towards a Th2 milieu (181). Vitamin D supplementation reduces IL1, IL6 and IL12 secretion by macrophages and downregulates TLR2 and TLR4 expression, while it enhances phagocytotic capacities (180). Similar results have been found for medroxyprogesterone, which could polarize monocytes into an M2 phenotype with downregulation of CD11c, IL1β and TNFα and upregulation of CD163 and IL10. Moreover, medroxyprogesterone could reverse M1 polarization and promoted decidualization and trophoblast invasion (182). For VEGF derived from decidual stromal cells, it has been established that it can upregulate M2 markers like CD206, CD163 and CCL17, and can reverse M1 polarization. It furthermore enhances macrophage migration (74). Another example is IL6 secreted by trophoblast, which activates the STAT-3 pathway and increases CD206, CCL18, IL10 and TGFβ in macrophages. Furthermore, these IL6 activated macrophages promoted trophoblast invasion and migration (77). Also hyaluronan can activate STAT-3 and -6 by binding to CD44 on dMφs. This downregulates M1-like receptors like CD80 and CD86, and upregulates CD163 and CD206 (78). In mice, human chorionic gonadotropin (hCG) administration has been shown to reduce the number of dMφs and induce M2 polarization (183).

A drug that could potentially downregulate the maternal inflammatory response in FGR is hydroxychloroquine, an antimalarial drug by origin. In pregnant women, hydroxychloroquine has been shown to ameliorate autoimmune diseases like systemic lupus erythematosus (SLE) and antiphospholipid syndrome (APS), and it has been associated with lower PE and preterm birth rates, and an increased birth weight (184). Its immune modulatory effects include decreasing the production of prostaglandins and pro-inflammatory cytokines like TNFα, IFNγ and IL6, inhibiting MMPs and blocking TRL signaling pathways (185, 186). Furthermore, it impairs antigen processing in antigen presenting cells and reduces T cell activation (177). Hydroxychloroquine can have a direct anti-inflammatory effect on first trimester trophoblast tissue in pregnant women with APS and excessive placental inflammation and insufficiency (187). Another agent to might have favorable effects in FGR is curcumin. It has been established that it can promote trophoblast growth, and reduce oxidative stress and the secretion of pro-inflammatory cytokines (e.g. IL6, IL8) in the placental villi and fetal membranes (188, 189). In rats, it has been shown to improve defects in trophoblast invasion and SA remodeling, along with a decrease in NFkβ, IL6 and MCP1 (190). In a rat PE model, curcumin combined with aspirin resulted in a reduction in placental oxidative stress, with decreased sFlt-1 and pro-inflammatory cytokines, and increased PlGF expression (191). Although not investigated in FGR specifically, its mechanism of action might improve placental function in FGR as well.

Prior to exploring therapies that directly affect macrophages, acquiring a deeper understanding of the pathways through which dMφs and HBCs affect placental functioning in FGR and other immune-mediated pregnancy complications is crucial. Moreover, the use of potential macrophage modulating therapies requires adequate diagnostic tools to determine the pathophysiological mechanism in individual FGR cases and personalize the therapeutic approach accordingly.

## 8 Conclusion

Our comprehension of the role of maternal and fetal macrophages in ensuring a successful pregnancy has significantly advanced. DMφs and HBCs are important in several processes during gestation, such as implantation of the embryo, remodeling of the uterine arteries, and safeguarding maternal-fetal tolerance. To carry out these functionalities, macrophages exhibit a wide range of phenotypical variation on a spectrum with pro- and anti-inflammatory characteristics at its opposite ends. Notably, this requires temporal regulation and quick adaption when needed. Dysregulation of macrophage subsets has been implicated in impaired placental functioning and compromised maternal-fetal tolerance. Subsequently, this has been linked to the development of various common pregnancy complications. Given the pathophysiological features of FGR, with impaired development of the placental vascularity and villous structure, leading to oxidative stress and inflammation, the involvement of macrophages in the genesis of these placental deficits seems evident. Although studies on macrophages in FGR specifically are scarce, existing literature in humans and mice shows that macrophages of a more pro-inflammatory character predominate over macrophages with immunomodulatory properties, compromising placental functioning and thereby fetal growth. More research dedicated to this topic is essential to obtain a broader understanding of the immunological component of the pathophysiology of FGR and work towards investigating potential therapies targeted at modulating the phenotype and functionality of dMφs and HBCs.

## Author contributions

RB: Data curation, Investigation, Writing – original draft. MF: Supervision, Writing – review & editing. HvG: Supervision, Writing – review & editing. SG: Supervision, Writing – review & editing. JP: Supervision, Writing – review & editing.

## References

[1] Baergen R. Manual of Pathology of the Human Placenta. New York: Springer Verlag New York Inc (2011).

[2] Yao Y Xu XH Jin L. Macrophage polarization in physiological and pathological pregnancy. Front Immunol. (2019) 10:792/full. doi: 10.3389/fimmu.2019.00792/full 31037072 PMC6476302

[3] Tang Z Abrahams VM Mor G Guller S. Placental Hofbauer cells and complications of pregnancy. Ann N Y Acad Sci. (2011) 1221:103–8. 10.1111/j.1749-6632.2010.05932.x PMC3707113 21401637

[4] Mor G Aldo P Alvero AB. The unique immunological and microbial aspects of pregnancy. Nat Rev Immunol. (2017) 17:469–82. doi: 10.1038/nri.2017.64 28627518

[5] Brown MB von Chamier M Allam AB Reyes L. M1/M2 macrophage polarity in normal and complicated pregnancy. Front Immunol. (2014) 5:1–10. doi: 10.3389/fimmu.2014.00606 PMC4241843 25505471

[6] Ygberg S Nilsson A. The developing immune system - From foetus to toddler. Acta Paediatrica Int J Paediatrics. (2012) 101:120–7. doi: 10.1111/j.1651-2227.2011.02494.x 22003882

[7] Maródi L. Innate cellular immune responses in newborns. Clin Immunol. (2006) 118:137–44. doi: 10.1016/j.clim.2005.10.012 16377252

[8] Zulu MZ Martinez FO Gordon S Gray CM. The elusive role of placental macrophages: the hofbauer cell. J Innate Immun. (2019), 447–56. doi: 10.1159/000497416 PMC6758944 30970346

[9] Khong TY Mooney EE Ariel I Balmus NCM Boyd TK Brundler MA. Sampling and definitions of placental lesions Amsterdam placental workshop group consensus statement. Arch Pathol Lab Med. (2016), 698–713. doi: 10.5858/arpa.2015-0225-CC 27223167

[10] Spinillo A Gardella B Adamo L Muscettola G Fiandrino G Cesari S. Pathologic placental lesions in early and late fetal growth restriction. Acta Obstet Gynecol Scand. (2019) 98:1585–94. doi: 10.1111/aogs.13699 31370094

[11] Burton GJ Jauniaux E. Pathophysiology of placental-derived fetal growth restriction. Am J Obstet Gynecol. (2018) 218:S745–61. doi: 10.1016/j.ajog.2017.11.577 29422210

[12] Junqueira LC CJ. Functionele histologie. 14th ed. Amsterdam: Amsterdam Reed Business Education (2014).

[13] Munoz-Suano A Hamilton AB Betz AG. Gimme shelter: the immune system during pregnancy. 10.1111/j.1600-065X.2011.01002.x 21488887

[14] Pijnenborg R Vercruysse L Hanssens M. The uterine spiral arteries in human pregnancy: Facts and controversies. Placenta. (2006) 27(9-10):939–68. 10.1016/j.placenta.2005.12.006 16490251

[15] Burton GJ Woods AW Jauniaux E Kingdom JCP. Rheological and physiological consequences of conversion of the maternal spiral arteries for uteroplacental blood flow during human pregnancy. Placenta. (2009) 30(6):473–82. 10.1016/j.placenta.2009.02.009 PMC2697319 19375795

[16] Nardozza LMM Caetano ACR Zamarian ACP Mazzola JB Silva CP Marçal VMG. Fetal growth restriction: current knowledge. Arch Gynecol Obstet. (2017) 295:1061–77. doi: 10.1007/s00404-017-4341-9 28285426

[17] Audette MC Kingdom JC. Screening for fetal growth restriction and placental insufficiency. Semin Fetal Neonatal Med. (2018) 23:119–25. doi: 10.1016/j.siny.2017.11.004 29221766

[18] Chauhan SP Beydoun H Chang E Sandlin AT Dahlke JD Igwe E. Prenatal detection of fetal growth restriction in newborns classified as small for gestational age: Correlates and risk of neonatal morbidity. Am J Perinatol. (2014) 31:187–94. 10.1055/s-0033-1343771 23592315

[19] Korteweg FJ Erwich JJHM Timmer A van der Meer J Ravisé JM Veeger NJGM. Evaluation of 1025 fetal deaths: proposed diagnostic workup. Am J Obstet Gynecol. (2011) 206:e1–12. 22196684 10.1016/j.ajog.2011.10.026

[20] Miller SL Huppi PS Mallard C. The consequences of fetal growth restriction on brain structure and neurodevelopmental outcome Fetal growth restriction Structural and functional deficits. J Physiol. (2016) 5944:807–23. doi: 10.1113/JP271402 PMC4753264 26607046

[21] Pallotto EK Kilbride HW. Perinatal outcome and later implications of intrauterine growth restriction. Clin Obstet Gynecol. (2006) 49:257–69. doi: 10.1097/00003081-200606000-00008 16721105

[22] Barker DJP. Adult consequences of fetal growth restriction. Clin Obstet Gynecol. (2006) 49:270–83. doi: 10.1097/00003081-200606000-00009 16721106

[23] Freedman AA Keenan-Devlin LS Borders A Miller GE Ernst LM. Formulating a meaningful and comprehensive placental phenotypic classification. Pediatr Dev Pathol. (2021) 24:337–50. doi: 10.1177/10935266211008444 PMC8277726 33872108

[24] Bezemer RE Schoots MH Timmer A Scherjon SA. Altered levels of decidual immune cell subsets in fetal growth restriction, stillbirth, and placental pathology. Front Immunol. (2020) 11:1–14. doi: 10.3389/fimmu.2020.01898 PMC7468421 32973787

[25] Schoots MH Gordijn SJ Scherjon SA van Goor H Hillebrands JL. Oxidative stress in placental pathology. Placenta. (2018) 69):153–61. doi: 10.1016/j.placenta.2018.03.003 29622278

[26] Redline RW Boyd T Campbell V Hyde S Kaplan C Khong TY. Maternal vascular underperfusion: nosology and reproducibility of placental reaction patterns. Pediatr Dev Pathol. (2004) 7:237–49. doi: 10.1007/s10024-003-8083-2 15022063

[27] Lyall F Robson SC Bulmer JN. Spiral artery remodeling and trophoblast invasion in preeclampsia and fetal growth restriction relationship to clinical outcome. Hypertension. (2013) 62:1046–54. doi: 10.1161/HYPERTENSIONAHA.113.01892 24060885

[28] Dunk C Kwan M Hazan A Walker S Wright JK Harris LK. Failure of decidualization and maternal immune tolerance underlies uterovascular resistance in intra uterine growth restriction. Front Endocrinol (Lausanne). (2019) 10:1–18. doi: 10.3389/fendo.2019.00160 PMC6436182 30949130

[29] Heider A. Fetal vascular malperfusion. Arch Pathol Lab Med. (2017) 141(11):1484–9. 10.5858/arpa.2017-0212-RA 29072954

[30] Redline RW Ravishankar S. Fetal vascular malperfusion, an update. APMIS. (2018) 126(7):561–9. 10.1111/apm.12849 30129125

[31] Umapathy A Chamley LW James JL. Reconciling the distinct roles of angiogenic/anti-angiogenic factors in the placenta and maternal circulation of normal and pathological pregnancies. Angiogenesis. (2020) 23:105–17. doi: 10.1007/s10456-019-09694-w 31707538

[32] Gusar VA Timofeeva AV Chagovets VV Vysokikh MY Kan NE Manukhova LA. Interrelation between miRNAs expression associated with redox state fluctuations, immune and inflammatory response activation, and neonatal outcomes in complicated pregnancy, accompanied by placental insufficienc. Antioxidants. (2023) 12. 10.3390/antiox12010006 PMC9854567 36670868

[33] Gusar VA Ganichkina M Chagovets VV Kan NE. MiRNAs regulating oxidative stress: A correlation with doppler sonography of uteroplacental complex and clinical state assessments of newborns in fetal growth restriction. J Clin Med. (2020) 9. 10.3390/jcm9103227 PMC7650709 33050114

[34] Redline RW. Villitis of unknown etiology: noninfectious chronic villitis in the placenta. Hum Pathol. (2007) 38(10):1439–46. 10.1016/j.humpath.2007.05.025 17889674

[35] Tamblyn JA Lissauer DM Powell R Coxb P Kilby MD. The immunological basis of villitis of unknown etiology - Review. Placenta. (2013) 34:846–55. doi: 10.1016/j.placenta.2013.07.002 23891153

[36] Sharps MC Baker BC Guevera T Bishof H Jones RL Greenwood SL. Increased placental macrophages and a pro-inflammatory profile in placentas and maternal serum in infants with a decreased growth rate in the third trimester of pregnancy. Am J Reprod Immunol. (2020), 1–14. doi: 10.1111/aji.13267 32421915

[37] Smith SC Baker PN Symonds EM. Increased placental apoptosis in intrauterine growth restriction. Am J Obstet Gynecol. (1997) 177:1395–401. doi: 10.1016/S0002-9378(97)70081-4 9423741

[38] Ishihara N Matsuo H Murakoshi H Laoag-Fernandez JB Samoto T Maruo T. Increased apoptosis in the syncytiotrophoblast in human term placentas complicated by either preeclampsia or intrauterine growth retardation. Am J Obstet Gynecol. (2002) 186:158–66. doi: 10.1067/mob.2002.119176 11810103

[39] Crocker IP Cooper S Ong SC Baker PN. Differences in apoptotic susceptibility of cytotrophoblasts and syncytiotrophoblasts in normal pregnancy to those complicated with preeclampsia and intrauterine growth restriction. Am J Pathol. (2003) 162:637–43. doi: 10.1016/S0002-9440(10)63857-6 PMC1851173 12547721

[40] Longtine MS Chen B Odibo AO Zhong Y Nelson DM. Villous trophoblast apoptosis is elevated and restricted to cytotrophoblasts in pregnancies complicated by preeclampsia, IUGR, or preeclampsia with IUGR. Placenta. (2021) 33:352–9. doi: 10.1016/j.placenta.2012.01.017 PMC3467099 22341340

[41] Redman CWG Sargent IL. Placental debris, oxidative stress and pre-eclampsia. Placenta. (2000) 21:597–602. doi: 10.1053/plac.2000.0560 10985960

[42] Chamley LW Chen Q Ding J Stone PR Abumaree M. Trophoblast deportation: Just a waste disposal system or antigen sharing? J Reprod Immunol. (2011) 88:99–105. doi: 10.1016/j.jri.2011.01.002 21334749

[43] Wang X Zhu H Lei L Zhang Y Tang C Wu JX. Integrated analysis of key genes and pathways involved in fetal growth restriction and their associations with the dysregulation of the maternal immune system. Front Genet. (2021) 11:5811789. doi: 10.3389/fgene.2020.581789 PMC7873903 33584788

[44] Majewska M Lipka A Paukszto L Jastrzebski JP Szeszko K Gowkielewicz M. Placenta transcriptome profiling in intrauterine growth restriction (IUGR). Int J Mol Sci. (2019) 20. doi: 10.3390/ijms20061510 PMC6471577 30917529

[45] McMinn J Wei M Schupf N Cusmai J Johnson EB Smith AC. Unbalanced placental expression of imprinted genes in human intrauterine growth restriction. Placenta. (2006) 27:540–9. doi: 10.1016/j.placenta.2005.07.004 16125225

[46] Greenbaum S Averbukh I Soon E Rizzuto G Baranski A Greenwald NF. A spatially resolved timeline of the human maternal-fetal interface. Nature. (2023) 619:595–605. doi: 10.1038/s41586-023-06298-9 37468587 PMC10356615

[47] Faas MM de Vos P. Uterine NK cells and macrophages in pregnancy. Placenta. (2017) 56:44–52. doi: 10.1016/j.placenta.2017.03.001 28284455

[48] Yang F Zheng Q Jin L. Dynamic function and composition changes of immune cells during normal and pathological pregnancy at the maternal-fetal interface. Front Immunol. (2019) 10:1–15. doi: 10.3389/fimmu.2019.02317 PMC6813251 31681264

[49] Mantovani A Sica A Sozzani S Allavena P Vecchi A Locati M. The chemokine system in diverse forms of macrophage activation and polarization. Trends Immunol. (2004) 25:677–86. doi: 10.1016/j.it.2004.09.015 15530839

[50] Nagamatsu T Schust DJ. The contribution of macrophages to normal and pathological pregnancies. Am J Reprod Immunol. (2010) 63:460–71. doi: 10.1111/j.1600-0897.2010.00813.x 20163399

[51] Parasar P Guru N Nayak NR. Contribution of macrophages to fetomaternal immunological tolerance. Hum Immunol. (2021) 82:325–31. doi: 10.1016/j.humimm.2021.02.013 PMC8062290 33715911

[52] Nagamatsu T Schust DJ. Review: the immunomodulatory roles of macrophages at the maternal-fetal interface. Reprod Sci. (2010) 17:209–18. doi: 10.1177/1933719109349962 20065301

[53] Svensson-Arvelund J Ernerudh J. The role of macrophages in promoting and maintaining homeostasis at the fetal-maternal interface. Am J Reprod Immunol. (2015) 74(2):100–9. 10.1111/aji.12357 25582625

[54] Jena MK Nayak N Chen K Nayak N. Role of macrophages in pregnancy and related complications. Arch Immunol Ther Exp. (2019) 67:295–309. doi: 10.1007/s00005-019-00552-7 PMC7140981 31286151

[55] Jaiswal MK Mallers TM Larsen B Kwak-Kim J Chaouat G Gilman-Sachs A. V-ATPase upregulation during early pregnancy: a possible link to establishment of an inflammatory response during preimplantation period of pregnancy. Reproduction. (2012) 143:713–25. doi: 10.1530/REP-12-0036 22454532

[56] Helige C Ahammer H Moser G Hammer A Dohr G Huppertz B. Distribution of decidual natural killer cells and macrophages in the neighbourhood of the trophoblast invasion front: a quantitative evaluation. Hum Reprod. (2014) 29:8–17. doi: 10.1093/humrep/det353 24140594

[57] Abrahams VM Visintin I Aldo PB Guller S Romero R Mor G. A role for TLRs in the regulation of immune cell migration by first trimester trophoblast cells. J Immunol. (2005) 175:8096–104. doi: 10.4049/jimmunol.175.12.8096 16339547

[58] Fest S Aldo PB Abrahams VM Visintin I Alvero A Chen R. Trophoblast-macrophage interactions: a regulatory network for the protection of pregnancy. Am J Reprod Immunol. (2007) 57:55–66. doi: 10.1111/j.1600-0897.2006.00446.x 17156192

[59] Drake PM Gunn MD Charo IF Tsou CL Zhou Y Huang L. Human Placental Cytotrophoblasts Attract Monocytes and CD56 bright Natural Killer Cells via the Actions of Monocyte Inflammatory Protein 1alfa. J Exp Med. (2001) 193:1199–212. doi: 10.1084/jem.193.10.1199 PMC2193324 11369791

[60] Mor G Romero R Aldo PB Abrahams VM. Is the trophoblast an immune regulator? The role of toll-like receptors during pregnancy. Crit Rev Immunol. (2005) 25:375–88. doi: 10.1615/CritRevImmunol.v25.i5 16167887

[61] Co EC Gormley M Kapidzic M Rosen DB Scott MA Stolp HAR. Maternal decidual macrophages inhibit NK cell killing of invasive cytotrophoblasts during human pregnancy. Biol Reprod. (2013) 88:1–9. doi: 10.1095/biolreprod.112.099465 23553431 PMC4070869

[62] Aschkenazi S Straszewski S Verwer KMA Foellmer H Rutherford T Mor G. Differential regulation and function of the Fas/Fas ligand system in human trophoblast cells. Biol Reprod. (2002) 66:1853–61. doi: 10.1095/biolreprod66.6.1853 12021072

[63] Ding J Yang C Zhang Y Wang J Zhang S Guo D. M2 macrophage-derived G-CSF promotes trophoblasts EMT, invasion and migration via activating PI3K/Akt/Erk1/2 pathway to mediate normal pregnancy. J Cell Mol Med. (2021) 25:2136–47. doi: 10.1111/jcmm.16191 PMC7882967 33393205

[64] Fock V Mairhofer M Otti GR Hiden U Spittler A Zeisler H. Macrophage-derived IL-33 is a critical factor for placental growth. J Immunol. (2013) 191:3734–43. doi: 10.4049/jimmunol.1300490 23997215

[65] Renaud SJ Postovit LM Macdonald-Goodfellow SK McDonald GT Caldwell JD Graham CH. Activated macrophages inhibit human cytotrophoblast invasiveness *in vitro* . Biol Reprod. (2005) 73:237–43. doi: 10.1095/biolreprod.104.038000 15800179

[66] Renaud SJ Macdonald-Goodfellow SK Graham CH. Coordinated regulation of human trophoblast invasiveness by macrophages and interleukin 101. Biol Reprod. (2007) 76:448–54. doi: 10.1095/biolreprod.106.055376 17151353

[67] Tian FJ Qin CM Li XC Wu F Liu XR Xu WM. Decreased stathmin-1 expression inhibits trophoblast proliferation and invasion and is associated with recurrent miscarriage. Am J Pathol. (2015) 185:2709–21. doi: 10.1016/j.ajpath.2015.06.010 26272359

[68] Buckley RJ Whitley GS Dumitriu IE Cartwright JE. Macrophage polarisation affects their regulation of trophoblast behaviour. Placenta. (2016) 47:73–80. doi: 10.1016/j.placenta.2016.09.004 27780542

[69] Kämmerer U Eggert AO Kapp M McLellan AD Geijtenbeek TBH Dietl J. Unique appearance of proliferating antigen-presenting cells expressing DC-SIGN (CD209) in the decidua of early human pregnancy. Am J Pathol. (2003) 162:887–96. doi: 10.1016/S0002-9440(10)63884-9 PMC1868095 12598322

[70] Gardner L Moffett A. Dendritic cells in the human decidua. Biol Reprod. (2003) 69:1438–46. doi: 10.1095/biolreprod.103.017574 12826583

[71] Houser BL Tilburgs T Hill J Nicotra ML Strominger JL. Two unique human decidual macrophage populations. J Immunol. (2011) 186:2633–42. doi: 10.4049/jimmunol.1003153 PMC3712354 21257965

[72] Svensson J Jenmalm MC Matussek A Geffers R Berg G Ernerudh J. Macrophages at the fetal–maternal interface express markers of alternative activation and are induced by M-CSF and IL-10. J Immunol. (2011) 187:3671–82. doi: 10.4049/jimmunol.1100130 21890660

[73] Svensson-Arvelund J Mehta RB Lindau R Mirrasekhian E Rodriguez-Martinez H Berg G. The human fetal placenta promotes tolerance against the semiallogeneic fetus by inducing regulatory T cells and homeostatic M2 macrophages. J Immunol. (2015) 194:1534–44. doi: 10.4049/jimmunol.1401536 25560409

[74] Wheeler KC Jena MK Pradhan BS Nayak N Das S Hsu CD. VEGF may contribute to macrophage recruitment and M2 polarization in the decidua. PLoS One. (2018) 13:1–18. doi: 10.1371/journal.pone.0191040 PMC5764356 29324807

[75] Laskarin G Cupurdija K Sotosek Tokmadzic V Dorcic D Dupor J Juretic K. The presence of functional mannose receptor on macrophages at the maternal-fetal interface. Hum Reprod. (2005) 20:1057–66. doi: 10.1093/humrep/deh740 15746201

[76] Gustafsson C Mjösberg J Matussek A Geffers R Matthiesen L Berg G. Gene expression profiling of human decidual macrophages: Evidence for immunosuppressive phenotype. PLoS One. (2008) 3:1–9. doi: 10.1371/journal.pone.0002078 PMC2323105 18446208

[77] Ding J Yang C Cheng Y Wang J Zhang S Yan S. Trophoblast-derived IL-6 serves as an important factor for normal pregnancy by activating Stat3-mediated M2 macrophages polarization. Int Immunopharmacol. (2020) 90:106788. doi: 10.1016/j.intimp.2020.106788 32718866

[78] Wang S Sun F Han M Liu Y Zou Q Wang F. Trophoblast-derived hyaluronan promotes the regulatory phenotype of decidual macrophages. Reproduction. (2019) 157:189–98. doi: 10.1530/REP-18-0450 30605433

[79] Petroff MG Sedlmayr P Azzola D Hunt JS. Decidual macrophages are potentially susceptible to inhibition by class Ia and class Ib HLA molecules. J Reprod Immunol. (2002) 56:3–17. doi: 10.1016/S0165-0378(02)00024-4 12106880

[80] Li C Houser BL Nicotra ML Strominger JL. HLA-G homodimer-induced cytokine secretion through HLA-G receptors on human decidual macrophages and natural killer cells. Proc Natl Acad Sci U.S.A. (2009) 106:5767–72. 10.1073/pnas.0901173106 PMC2667005 19304799

[81] Lombardelli L Aguerre-Girr M Logiodice F Kullolli O Casart Y Polgar B. HLA-G5 induces IL-4 secretion critical for successful pregnancy through differential expression of ILT2 receptor on decidual CD4 + T cells and macrophages. J Immunol. (2013) 191:3651–62. doi: 10.4049/jimmunol.1300567 23997222

[82] Lee CL Guo Y So KH Vijayan M Guo Y Wong VHH. Soluble human leukocyte antigen G5 polarizes differentiation of macrophages toward a decidual macrophage-like phenotype. Hum Reprod. (2015) 30:2263–74. doi: 10.1093/humrep/dev196 26307092

[83] Murray AJ. Oxygen delivery and fetal-placental growth: Beyond a question of supply and demand? Placenta. (2012) 33:e16–22. doi: 10.1016/j.placenta.2012.06.006 22742726

[84] Harris LK Benagiano M D’Elios MM Brosens I Benagiano G. Placental bed research: II. Functional and immunological investigations of the placental bed. Am J Obstet Gynecol. (2019) 221:457–69. doi: 10.1016/j.ajog.2019.07.010 31288009

[85] Smith S Dunk C Aplin J Harris L Jones R. Evidence for immune cell involvement in decidual spiral arteriole remodeling in early human pregnancy. Am J Pathol. (2009) 174:1959–71. doi: 10.2353/ajpath.2009.080995 PMC2671283 19349361

[86] Hazan AD Smith SD Jones RL Whittle W Lye SJ Dunk CE. Vascular-leukocyte interactions: mechanisms of human decidual spiral artery remodeling in *vitro* . Am J Pathol. (2010) 177:1017–30. doi: 10.2353/ajpath.2010.091105 PMC2913364 20558572

[87] Imanishi T Hano T Nishio I Han DKM Schwartz SM. Erratum: Apoptosis of vascular smooth muscle cells is induced by Fas ligand derived from endothelial cells [Japan Circulation Journal (2001) 65(556–560)]. Circ J. (2002) 66:1185. 10.1253/jcj.65.556 11407740

[88] Abrahams V Kim Y Straszewski S Romero R Mor G. Macrophages and apoptotic cell clearance during pregnancy. Am J Reprod Immunol. (2004) 51:275–82. doi: 10.1111/j.1600-0897.2004.00156.x 15212680

[89] Clark DE Smith SK Licence D Evans AL Charnock-Jones DS. Comparison of expression patterns for placenta growth factor, vascular endothelial growth factor (VEGF), VEGF-B and VEGF-C in the human placenta throughout gestation. J Endocrinol. (1998) 159:459–67. doi: 10.1677/joe.0.1590459 9834463

[90] Lash GE Pitman H Morgan HL Innes BA Agwu CN Bulmer JN. Decidual macrophages: key regulators of vascular remodeling in human pregnancy. J Leukoc Biol. (2016) 100:315–25. doi: 10.1189/jlb.1A0815-351R 26819320

[91] Gill N Leng Y Romero R Xu Y Panaitescu B Miller D. The immunophenotype of decidual macrophages in acute atherosis. Am J Reprod Immunol. (2019) 81:e13098. doi: 10.1111/aji.13098 30734977 PMC6556389

[92] Kaufmann P Black S Huppertz B. Endovascular trophoblast invasion: Implications for the pathogenesis of intrauterine growth retardation and preeclampsia. Biol Reprod. (2003) 69:1–7. doi: 10.1095/biolreprod.102.014977 12620937

[93] Fernekorn U Butcher EC Behrends J Hartz S Kruse A. Functional involvement of P-selectin and MAdCAM-1 in the recruitment of α4β7-integrin-expressing monocyte-like cells to the pregnant mouse uterus. Eur J Immunol. (2004) 34:3423–33. doi: 10.1002/eji.200425223 15484189

[94] Care AS Diener KR Jasper MJ Brown HM Ingman WV Robertson SA. Macrophages regulate corpus luteum development during embryo implantation in mice. J Clin Invest. (2013) 123:3472–87. doi: 10.1172/JCI60561 PMC3726148 23867505

[95] Miwa N Hayakawa S Miyazaki S Myojo S Sasaki Y Sakai M. IDO expression on decidual and peripheral blood dendritic cells and monocytes/macrophages after treatment with CTLA-4 or interferon-γ increase in normal pregnancy but decrease in spontaneous abortion. Mol Hum Reprod. (2006) 11:865–70. 10.1093/molehr/gah246 16421220

[96] Sayama S Nagamatsu T Schust DJ Itaoka N Ichikawa M Kawana K. Human decidual macrophages suppress IFN-γ production by T cells through costimulatory B7-H1: PD-1 signaling in early pregnancy. J Reprod Immunol. (2013) 100:109–17. doi: 10.1016/j.jri.2013.08.001 24045115

[97] Repnik U Tilburgs T Roelen DL van der Mast BJ Kanhai HHH Scherjon S. Comparison of macrophage phenotype between decidua basalis and decidua parietalis by flow cytometry. Placenta. (2008) 29:405–12. doi: 10.1016/j.placenta.2008.02.004 18353434

[98] Wang H He M Hou Y Chen S Zhang X Zhang M. Role of decidual CD14+ macrophages in the homeostasis of maternal-fetal interface and the differentiation capacity of the cells during pregnancy and parturition. Placenta. (2016) 38:76–83. doi: 10.1016/j.placenta.2015.12.001 26907385

[99] Rajakumar A Kane MA Yu J Jones JW Qu H Badell M. Alternatively activated macrophages are the primary retinoic acid-producing cells in human decidua. Reprod Sci. (2020) 27:334–41. doi: 10.1007/s43032-019-00030-7 PMC7539807 32046391

[100] Heikkinen J Möttönen M Komi J Alanen A Lassila O. Phenotypic characterization of human decidual macrophages. Clin Exp Immunol. (2003) 131:498–505. doi: 10.1046/j.1365-2249.2003.02092.x 12605704 PMC1808648

[101] Kim SY Romero R Tarca AL Bhatti G Kim CJ Lee J. Methylome of fetal and maternal monocytes and macrophages at the feto-maternal interface. (2013) 68(1):8–27. 10.1111/j.1600-0897.2012.01108.x PMC3479407 22385097

[102] Lindau R Vondra S Spreckels J Solders M Svensson-Arvelund J Berg G. Decidual stromal cells support tolerance at the human foetal-maternal interface by inducing regulatory M2 macrophages and regulatory T-cells. J Reprod Immunol. (2021) 146:103330. doi: 10.1016/j.jri.2021.103330 34049032

[103] Su L Sun Y Ma F Lü P Huang H Zhou J. Progesterone inhibits Toll-like receptor 4-mediated innate immune response in macrophages by suppressing NF-κB activation and enhancing SOCS1 expression. Immunol Lett. (2009) 125:151–5. doi: 10.1016/j.imlet.2009.07.003 19607861

[104] Abumaree MH Chamley LW Badri M El-Muzaini MF. Trophoblast debris modulates the expression of immune proteins in macrophages: A key to maternal tolerance of the fetal allograft? J Reprod Immunol. (2012) 94:131–41. doi: 10.1016/j.jri.2012.03.488 22542910

[105] Osman I Young A Ledingham MA Thomson AJ Jordan F Greer IA. Leukocyte density and pro-inflammatory cytokine expression in human fetal membranes, decidua, cervix and myometrium before and during labour at term. Mol Hum Reprod. (2003) 9:41–5. doi: 10.1093/molehr/gag001 12529419

[106] Xu Y Romero R Miller D Kadam L Mial T Plaszyo O. An M1-like macrophage polarization in decidual tissue during spontaneous preterm labor that is attenuated by rosiglitazone treatment. J Immunol. (2016) 196:2476–91. doi: 10.4049/jimmunol.1502055 PMC4779725 26889045

[107] Hamilton S Oomomian Y Stephen G Shynlova O Tower CL Garrod A. Macrophages infiltrate the human and rat decidua during term and preterm labor: Evidence that decidual inflammation precedes labor1. Biol Reprod. (2011) 86:1–9. 10.1095/biolreprod.111.095505 22011391

[108] Ingman K Cookson VJKW Jones CJP Aplin JD. Characterisation of hofbauer cells in first and second trimester placenta: Incidence, phenotype, survival *in vitro* and motility. Placenta. (2010) 31:535–44. doi: 10.1016/j.placenta.2010.03.003 20347485

[109] Vinnars MTN Rindsjö E Ghazi S Sundberg A Papadogiannakis N. The number of CD68+ (Hofbauer) cells is decreased in placentas with chorioamnionitis and with advancing gestational age. Pediatr Dev Pathol. (2010) 13:300–4. doi: 10.2350/09-03-0632-OA.1 19642814

[110] Swieboda D Johnson E Beaver J Haddad L Enninga EAL Hathcock M. Baby’s first MФ: temporal regulation of hofbauer cell phenotype influences ligand-mediated innate immune responses across gestation. J Immunol. (2020) 204:2380–91. doi: 10.4049/jimmunol.1901185 PMC7870092 32213562

[111] Thomas J Appios A Zhao X Dutkiewicz R Donde M Lee C. Phenotypic and functional characterisation of first trimester human placental macrophages, Hofbauer cells. bioRxiv. (2020) 218. doi: 10.1101/2020.09.03.279919 PMC7579740 33075123

[112] Reyes L Golos TG. Hofbauer cells: Their role in healthy and complicated pregnancy. Front Immunol. (2018). 10.3389/fimmu.2018.02628 PMC6249321 30498493

[113] Yuan V Hui D Yin Y Peñaherrera MS Beristain AG Robinson WP. Cell-specific characterization of the placental methylome. BMC Genomics. (2021) 22:1–20. doi: 10.1186/s12864-020-07186-6 33407091 PMC7788826

[114] Mezouar S Katsogiannou M Ben A Bretelle F. Placental macrophages: Origin, heterogeneity, function and role in pregnancy-associated infections. (2021) 103(January):94–103. 10.1016/j.placenta.2020.10.017 PMC7568513 33120051

[115] Loegl J Hiden U Nussbaumer E Schliefsteiner C Cvitic S Lang I. Hofbauer cells of M2a, M2b and M2c polarization may regulate feto-placental angiogenesis. Reproduction. (2016) 152:447–55. doi: 10.1530/REP-16-0159 27534571

[116] Martinez FO Gordon S. The M1 and M2 paradigm of macrophage activation: Time for reassessment. F1000Prime Rep. (2014) 6:1–13. doi: 10.12703/P 24669294 PMC3944738

[117] Thomas JR Naidu P Appios A McGovern N. The ontogeny and function of placental macrophages. Front Immunol. (2021) 12. doi: 10.3389/fimmu.2021.771054 PMC8566952 34745147

[118] Bulmer JN Morrison L Smith JC. Expression of class II MHC gene products by macrophages in human uteroplacental tissue. Immunotogy. (1988) 63. PMC1454811 3284818

[119] Appios A Thomas JR McGovern N. Isolation of first-trimester and full-term human placental Hofbauer cells. Bio Protoc. (2021) 11. doi: 10.21769/BioProtoc.4044 PMC8250382 34250210

[120] Schliefsteiner C Ibesich S Wadsack C. Placental hofbauer cell polarization resists inflammatory cues in *vitro* . Int J Mol Sci. (2020) 21. doi: 10.3390/ijms21030736 PMC7038058 31979196

[121] Pavlov OV Selutin AV Pavlova OM Selkov SA. Two patterns of cytokine production by placental macrophages. Placenta. (2020) 91:1–10. doi: 10.1016/j.placenta.2020.01.005 31941612

[122] Liu Y Fan X Wang R Lu X Dang YL Wang H. Single-cell RNA-seq reveals the diversity of trophoblast subtypes and patterns of differentiation in the human placenta. Cell Res. (2018) 28:819–32. doi: 10.1038/s41422-018-0066-y PMC6082907 30042384

[123] Wood G King C. Trapping antigen-antibody complexes within the human placenta. Cell Immunol. (1982) 69:347–62. doi: 10.1016/0008-8749(82)90077-6 7049408

[124] Bright NA Ockleford CD Anwar M. Ontogeny and distribution of Fc gamma receptors in the human placenta. Transport or immune surveillance? J Anat. (1994) 184:297–308. 8014121 PMC1259990

[125] Palmeira P Quinello C Silveira-Lessa AL Zago CA Carneiro-Sampaio M. IgG placental transfer in healthy and pathological pregnancies. Clin Dev Immunol. (2012) 2012. doi: 10.1155/2012/985646 PMC3251916 22235228

[126] Seval Y Korgun ET Demir R. Hofbauer cells in early human placenta: Possible implications in vasculogenesis and angiogenesis. Placenta. (2007) 28:841–5. doi: 10.1016/j.placenta.2007.01.010 17350092

[127] Demir R Erbengi T. Some new findings about Hofbauer cells in the chorionic villi of the human placenta. Acta Anat (Basel). (1984) 199:18–26. doi: 10.1159/000145857 6730891

[128] Khan S Katabuchi H Araki M Nishimura R Okamura H. Human villous macrophage-conditioned media enhance human trophoblast growth and differentiation *in vitro* . Biol Reprod. (2000) 62:1075–83. doi: 10.1095/biolreprod62.4.1075 10727280

[129] Anteby EY Natanson-Yaron S Greenfield C Goldman-Wohl D Haimov-Kochman R Holzer H. Human placental Hofbauer cells express sprouty proteins: A possible modulating mechanism of villous branching. Placenta. (2005) 26:476–83. doi: 10.1016/j.placenta.2004.08.008 15950061

[130] Demir R Kayisli UA Seval Y Celik-Ozenci C Korgun ET Demir-Weusten AY. Sequential expression of VEGF and its receptors in human placental villi during very early pregnancy: Differences between placental vasculogenesis and angiogenesis. Placenta. (2004) 25:560–72. doi: 10.1016/j.placenta.2003.11.011 15135240

[131] Ahmed A Li XL Dunk C Whittle MJ Rushton I Rollason T. Colocalisation of vascular endothelial growth factor and its flt-1 receptor in human placenta. Growth Factors. (1995) 12. doi: 10.3109/08977199509036883 8619929

[132] Demir R. Expression of VEGF receptors VEFGR-1 and VEGFR-2, angiopoietin receptors Tie-1 and Tie-2 in chorionic villi tree during early pregnancy. Folia Histochem Cytobiol. (2009) 47:435–45. 10.2478/v10042-009-0100-5 20164029

[133] Geva E Ginzinger DG Zaloudek CJ Moore DH Byrne A Jaffe RB. Human placental vascular development: Vasculogenic and angiogenic (branching and nonbranching) transformation is regulated by vascular endothelial growth factor-A, angiopoietin-1, and angiopoietin-2. J Clin Endocrinol Metab. (2002) 87:4213–24. doi: 10.1210/jc.2002-020195 12213874

[134] Pavlov OV Niauri DA Selutin AV Selkov SA. Coordinated expression of TNFα- and VEGF-mediated signaling components by placental macrophages in early and late pregnancy. Placenta. (2016) 42:28–36. doi: 10.1016/j.placenta.2016.04.008 27238711

[135] Johnson GA Burghardt RC Bazer FW Spencer TE. Osteopontin: roles in implantation and placentation. Biol Reprod. (2003) 69:1458–71. doi: 10.1095/biolreprod.103.020651 12890718

[136] Matsubara S Takayama T Iwasaki R Komatsu N Matsubara D Takizawa T. Enzyme-cytochemically detectable glucose-6-phosphate dehydrogenase in human villous macrophages (Hofbauer cells). Placenta. (2001) 22:882–5. doi: 10.1053/plac.2001.0720 11718577

[137] Martinoli C Castellucci M Zaccheo D Kaufmann P. Scanning electron microscopy of stromal cells of human placental villi throughout pregnancy. Cell Tissue Res. (1984) 235:647–55. doi: 10.1007/BF00226964 6713492

[138] Böckle BC Sölder E Kind S Romani N Sepp NT. DC-SIGN+ CD163+ Macrophages expressing hyaluronan receptor LYVE-1 are located within chorion villi of the placenta. Placenta. (2008) 29:187–92. doi: 10.1016/j.placenta.2007.11.003 18078989

[139] Young OM Tang Z Niven-Fairchild T Tadesse S Krikun G Norwitz ER. Toll-like receptor-mediated responses by placental hofbauer cells (HBCs): A potential pro-inflammatory role for fetal M2 macrophages. Am J Reprod Immunol. (2015) 73:22–35. doi: 10.1111/aji.12336 25345551 PMC4268350

[140] Tang Z Niven-Fairchild T Tadesse S Norwitz ER Buhimschi CS Buhimschi IA. Glucocorticoids enhance CD163 expression in placental hofbauer cells. Endocrinology. (2013) 154:471–82. doi: 10.1210/en.2012-1575 PMC3529384 23142809

[141] Pavlov O Pavlova O Ailamazyan E Selkov S. Characterization of cytokine production by human term placenta macrophages *in vitro* . Am J Reprod Immunol. (2008) 60:556–67. doi: 10.1111/j.1600-0897.2008.00657.x 18853988

[142] Goldstein J Braverman M Salafia C Buckley P. The phenotype of human placental macrophages and its variation with gestational age. Am J Pathol. (1988) 133:648–59. PMC1880826 3264459

[143] Selkov SA Selutin AV Pavlova OM Khromov-Borisov NN Pavlov OV. Comparative phenotypic characterization of human cord blood monocytes and placental macrophages at term. Placenta. (2013) 34:836–9. doi: 10.1016/j.placenta.2013.05.007 23773857

[144] Kudo Y Boyd CAR Spyropoulou I Redman CWG Takikawa O Katsuki T. Indoleamine 2,3-dioxygenase: Distribution and function in the developing human placenta. J Reprod Immunol. (2004) 61:87–98. doi: 10.1016/j.jri.2003.11.004 15063632

[145] Uren SJ Boyle W. Class II MHC antigen-positive macrophages from human placentae suppress strong MLR and CML reactions. Cell Immunol. (1990) 125:235–46. doi: 10.1016/0008-8749(90)90077-5 2136719

[146] Phillips TA Ni J Hunt JS. Death-inducing tumour necrosis factor (TNF) superfamily ligands and receptors are transcribed in human placentae, cytotrophoblasts, placental macrophages and placental cell lines. Placenta. (2001) 22:663–72. doi: 10.1053/plac.2001.0703 11597186

[147] Myatt L Eis ALW Brockman DE Kossenjans W Greer I Lyall F. Inducible (Type II) nitric oxide synthase in human placental villous tissue of normotensive, pre-eclamptic and intrauterine growth-restricted pregnancies. Placenta. (1997) 18:261–8. doi: 10.1016/S0143-4004(97)80060-4 9179919

[148] Wetzka B Clark D Charnock-Jones D Zahradnik H Smith S. Isolation of macrophages (Hofbauer cells) from human term placenta and their prostaglandin E2 and thromboxane production. Hum Reprod. (1997) 12:847–52. doi: 10.1093/humrep/12.4.847 9159455

[149] Pongcharoen S Somran J Sritippayawan S Niumsup P Chanchan P Butkhamchot P. Interleukin-17 expression in the human placenta. Placenta. (2007) 28:59–63. doi: 10.1016/j.placenta.2006.01.016 16549200

[150] Brosens I Pijnenborg R Vercruysse L Romero R. The “great Obstetrical Syndromes” are associated with disorders of deep placentation. Am J Obstetrics Gynecology. (2011), 193–201. doi: 10.1016/j.ajog.2010.08.009 PMC3369813 21094932

[151] Wang WJ Hao CF Lin QD. Dysregulation of macrophage activation by decidual regulatory T cells in unexplained recurrent miscarriage patients. J Reprod Immunol. (2011) 92:97–102. doi: 10.1016/j.jri.2011.08.004 22015003

[152] Schonkeren D van der Hoorn ML Khedoe P Swings G Van Beelen E Claas F. Differential distribution and phenotype of decidual macrophages in preeclamptic versus control pregnancies. Am J Pathol. (2011) 178:709–17. doi: 10.1016/j.ajpath.2010.10.011 PMC3069820 21281803

[153] Mifsud W Sebire NJ. Placental pathology in early-onset and late-onset fetal growth restriction. Fetal Diagnosis Ther. (2014) 36(2):117–28. 10.1159/000359969 24577279

[154] Williams PJ Bulmer JN Searle RF Innes BA Robson SC. Altered decidual leucocyte populations in the placental bed in pre-eclampsia and foetal growth restriction: a comparison with late normal pregnancy. Reproduction. (2009) 138:177–84. doi: 10.1530/REP-09-0007 19357130

[155] Chu A Najafzadeh P Sullivan P Cone B Janzen C Mah V. Aldehyde dehydrogenase isoforms and inflammatory cell populations are differently expressed in term human placentas affected by intrauterine growth restriction. Placenta. (2019) 81):9–17. doi: 10.1016/j.placenta.2019.03.015 31138432 PMC6719708

[156] Berezhna VA Mamontova TV Gromova AM. CD68+ M1 macrophages is associated with placental insufficiency under fetal growth restriction. Wiadomosci Lekarskie. (2021) 74:213–9. doi: 10.36740/WiadLek 33813474

[157] Li P Zhao Y. Integrative analysis of the immune - related ceRNA network in fetal growth restriction based on weighted gene co - expression network analysis. Arch Gynecol Obstet. (2022) 0123456789). doi: 10.1007/s00404-022-06805-9 36217035

[158] Umapathy A McCall A Sun C Boss AL Gamage TKJB Brooks AES. Mesenchymal stem/stromal cells from placentae of growth restricted pregnancies are poor stimulators of angiogenesis. Stem Cell Rev Rep. (2020) 16:557–68. doi: 10.1007/s12015-020-09959-8 32080795

[159] Dang Y Souchet C Moresi F Jeljeli M Raquillet B Nicco C. BCG-trained innate immunity leads to fetal growth restriction by altering immune cell profile in the mouse developing placenta. J Leukoc Biol. (2022) 111:1009–20. doi: 10.1002/JLB.4A0720-458RR 34533228

[160] Grigoriadis C Tympa A Creatsa M Bakas P Liapis A Kondi-Pafiti A. Hofbauer cells morphology and density in placentas from normal and pathological gestations. Rev Bras Ginecologia e Obstetrícia. (2013) 35:407–12. doi: 10.1590/S0100-72032013000900005 24217569

[161] Schmidt A Morales-Prieto DM Pastuschek J Fröhlich K Markert UR. Only humans have human placentas: Molecular differences between mice and humans. J Reprod Immunol. (2015) 108:65–71. doi: 10.1016/j.jri.2015.03.001 25817465

[162] Girardi G Yarilin D Thurman JM Holers VM Salmon JE. Complement activation induces dysregulation of angiogenic factors and causes fetal rejection and growth restriction. J Exp Med. (2006) 203:2165–75. doi: 10.1084/jem.20061022 PMC2118387 16923853

[163] An X Qin J Hu X Zhou Y Fu B Wei H. Overexpression of lipocalin 2 in PBX1-deficient decidual NK cells promotes inflammation at thematernal-fetal interface. Am J Reproduc. (2023) 89:e13676. 10.1111/aji.13676 36621850

[164] Brien ME Duval C Palacios J Boufaied I Hudon-Thibeault AA Nadeau-Vallée M. Uric acid crystals induce placental inflammation and alter trophoblast function via an IL-1–dependent pathway: Implications for fetal growth restriction. J Immunol. (2017) 198:443–51. doi: 10.4049/jimmunol.1601179 PMC5176081 27903743

[165] McKelvey KJ Yenson VM Ashton AW Morris JM McCracken SA. Embryonic/fetal mortality and intrauterine growth restriction is not exclusive to the CBA/J sub-strain in the CBA × DBA model. Sci Rep. (2016) 6:1–11. doi: 10.1038/srep35138 27767070 PMC5073309

[166] Ushida T Cotechini T Protopapas N Atallah A Collyer C Toews AJ. Aberrant inflammation in rat pregnancy leads to cardiometabolic alterations in the offspring and intrauterine growth restriction in the F2 generation. (2022). 10.1017/S2040174422000265 35593438

[167] Kaur G Porter CBM Ashenberg O Lee J Riesenfeld SJ Hofree M. Mouse fetal growth restriction through parental and fetal immune gene variation and intercellular communications cascade. Nat Commun. (2022) 13. doi: 10.1038/s41467-022-32171-w PMC9338297 35906236

[168] Melamed N Baschat A Yinon Y Athanasiadis A Mecacci F Figueras F. FIGO (international Federation of Gynecology and obstetrics) initiative on fetal growth: best practice advice for screening, diagnosis, and management of fetal growth restriction. Int J Gynecology Obstetrics. (2021) 152:3–57. doi: 10.1002/ijgo.13522 PMC8252743 33740264

[169] Schoots MH Bourgonje MF Bourgonje AR Prins JR van Hoorn EGM Abdulle AE. Oxidative stress biomarkers in fetal growth restriction with and without preeclampsia. Placenta. (2021) 115:87–96. doi: 10.1016/j.placenta.2021.09.013 34583270

[170] Fadigas C Peeva G Mendez O Poon LC Nicolaides KH. Prediction of small-for-gestational-age neonates: Screening by placental growth factor and soluble fms-like tyrosine kinase-1 at 35–37 weeks. Ultrasound Obstetrics Gynecology. (2015) 46:191–7. doi: 10.1002/uog.14862 25825848

[171] Ciobanu A Rouvali A Syngelaki A Akolekar R Nicolaides KH. Prediction of small for gestational age neonates: screening by maternal factors, fetal biometry, and biomarkers at 35–37 weeks’ gestation. Am J Obstet Gynecol. (2019) 220:486.e1–486.e11. doi: 10.1016/j.ajog.2019.01.227 30707967

[172] Bujold E Roberge S Lacasse Y Bureau M Audibert F Marcoux S. Prevention of preeclampsia and intrauterine growth restriction with aspirin started in early pregnancy. Obstetrics Gynecology. (2010) 116:402–14. doi: 10.1097/AOG.0b013e3181e9322a 20664402

[173] Roberge S Nicolaides K Demers S Hyett J Chaillet N Bujold E. The role of aspirin dose on the prevention of preeclampsia and fetal growth restriction: systematic review and meta-analysis. Am J Obstet Gynecol. (2017) 216:110–120.e6. 27640943 10.1016/j.ajog.2016.09.076

[174] Loussert L Vidal F Parant O Hamdi SM Vayssiere C Guerby P. Aspirin for prevention of preeclampsia and fetal growth restriction. Prenat Diagn. (2020) 40:519–27. doi: 10.1002/pd.5645 31955436

[175] Rambaldi MP Weiner E Mecacci F Bar J Petraglia F. Immunomodulation and preeclampsia. Best Pract Res Clin Obstet Gynaecol. (2019) 60:87–96. doi: 10.1016/j.bpobgyn.2019.06.005 31311760

[176] McLaughlin K Drewlo S Parker JD Kingdom JCP. Current theories on the prevention of severe preeclampsia with low-molecular weight heparin. Hypertension. (2015) 66:1098–103. doi: 10.1161/HYPERTENSIONAHA.115.05770 26441469

[177] Collier A Smith L Karumanchi S. Review of the immune mechanisms of preeclampsia and the potential immune modulating therapy. Hum Immunol. (2021) 82:362–70. doi: 10.1016/j.humimm.2021.01.004 PMC8062309 33551128

[178] Meijerink L Wever KE Terstappen F Ganzevoort W Lely AT Depmann M. Statins in pre-eclampsia or fetal growth restriction: A systematic review and meta-analysis on maternal blood pressure and fetal growth across species. BJOG. (2023) 130:577–85. doi: 10.1111/1471-0528.17393 36681887

[179] Pels A Kenny LC Alfirevic Z Baker PN von Dadelszen P Gluud C. STRIDER (Sildenafil TheRapy in dismal prognosis early onset fetal growth restriction): An international consortium of randomised placebo-controlled trials. BMC Pregnancy Childbirth. (2017) 17:1–8. doi: 10.1186/s12884-017-1594-z 29282009 PMC5745923

[180] Cyprian F Lefkou E Varoudi K Girardi G. Immunomodulatory effects of vitamin D in pregnancy and beyond. Front Immunol. (2019) 10:1–17. doi: 10.3389/fimmu.2019.02739 PMC6883724 31824513

[181] Shin J Choi M Longtine M Nelson D. Vitamin D effects on pregnancy and the placenta. Placenta. (2004) 31:1027–34. doi: 10.1016/j.placenta.2010.08.015 PMC2993775 20863562

[182] Tsai YC Tseng JT Wang CY Su MT Huang JY Kuo PL. Medroxyprogesterone acetate drives M2 macrophage differentiation toward a phenotype of decidual macrophage. Mol Cell Endocrinol. (2017) 452:74–83. doi: 10.1016/j.mce.2017.05.015 28522271

[183] Furcron AE Romero R Mial TN Balancio A Panaitescu B Hassan SS. Human chorionic gonadotropin has anti-inflammatory effects at the maternal-fetal interface and prevents endotoxin-induced preterm birth, but causes dystocia and fetal compromise in mice. Biol Reprod. (2016) 94:1–13. doi: 10.1095/biolreprod.116.139345 PMC4946806 27146032

[184] Seo MR Chae J Kim YM Cha HS Choi SJ Oh S. Hydroxychloroquine treatment during pregnancy in lupus patients is associated with lower risk of preeclampsia. Lupus. (2019) 28:722–30. doi: 10.1177/0961203319843343 30971164

[185] Arachchillage DJ Laffan M Pericleous C. Hydroxychloroquine as an immunomodulatory and antithrombotic treatment in antiphospholipid syndrome. Int J Mol Sci. (2023) 24. doi: 10.3390/ijms24021331 PMC9866802 36674847

[186] de Moreuil C Alavi Z Pasquier E. Hydroxychloroquine may be beneficial in preeclampsia and recurrent miscarriage. Br J Clin Pharmacol. (2020) 86:39–49. doi: 10.1111/bcp.14131 31633823 PMC6983516

[187] Albert CR Schlesinger WJ Viall CA Mulla MJ Brosens JJ Chamley LW. Effect of hydroxychloroquine on antiphospholipid antibody-induced changes in first trimester trophoblast function. Am J Reprod Immunol. (2014) 71:154–64. doi: 10.1111/aji.12184 24325143

[188] Wang Y Li B Zhao Y. Inflammation in preeclampsia: Genetic biomarkers, mechanisms, and therapeutic strategies. Front Immunol. (2022) 13:1–14. doi: 10.3389/fimmu.2022.883404 PMC9307876 35880174

[189] Lim R Barker G Wall CA Lappas M. Dietary phytophenols curcumin, naringenin and apigenin reduce infection-induced inflammatory and contractile pathways in human placenta, foetal membranes and myometrium. Mol Hum Reprod. (2013) 19:451–62. doi: 10.1093/molehr/gat015 23475986

[190] Gong P Liu M Hong G Li Y Xue P Zheng M. Curcumin improves LPS-induced preeclampsia-like phenotype in rat by inhibiting the TLR4 signaling pathway. Placenta. (2016) 41:45–52. doi: 10.1016/j.placenta.2016.03.002 27208407

[191] Ju Y Feng Y Yang Y Hou X Zhang X Zhu X. Combining curcumin and aspirin ameliorates preeclampsia-like symptoms by inhibiting the placental TLR4/NF-κB signaling pathway in rats. J Obstetrics Gynaecology Res. (2023) 49:128–40. doi: 10.1111/jog.15473 36288911

